# Biomineralization-inspired mineralized hydrogel promotes the repair and regeneration of dentin/bone hard tissue

**DOI:** 10.1038/s41536-023-00286-3

**Published:** 2023-02-25

**Authors:** Bo Wen, Yuguo Dai, Xue Han, Fangjun Huo, Li Xie, Mei Yu, Yuru Wang, Ning An, Zhonghan Li, Weihua Guo

**Affiliations:** 1 State Key Laboratory of Oral Disease & National Clinical Research Center for Oral Diseases, West China Hospital of Stomatology, Sichuan University, Chengdu, China; 2 National Engineering Laboratory for Oral Regenerative Medicine, West China Hospital of Stomatology, Sichuan University, Chengdu, China; 3 Department of Pediatric Dentistry, West China Hospital of Stomatology, Sichuan University, Chengdu, China; 4 Stomatological Hospital, School of Stomatology, Southern Medical University, Guangzhou, China; 5 Center of Growth Metabolism and Aging, Key Laboratory of Bio-Resource and Eco-Environment of Ministry of Education, College of Life Sciences, Sichuan University, Chengdu, China; 6 Yunnan Key Laboratory of Stomatology, The Affiliated Hospital of Stomatology, School of Stomatology, Kunming Medical University, Kunming, China

**Keywords:** Regenerative medicine, Biomedical materials, Biomaterials - cells

## Abstract

Maxillofacial hard tissue defects caused by trauma or infection often affect craniofacial function. Taking the natural hard tissue structure as a template, constructing an engineered tissue repair module is an important scheme to realize the functional regeneration and repair of maxillofacial hard tissue. Here, inspired by the biomineralization process, we constructed a composite mineral matrix hydrogel PAA-CMC-TDM containing amorphous calcium phosphates (ACPs), polyacrylic acid (PAA), carboxymethyl chitosan (CMC) and dentin matrix (TDM). The dynamic network composed of *Ca*^*2+*^*·COO*^*−*^ coordination and ACPs made the hydrogel loaded with TDM, and exhibited self-repairing ability and injectability. The mechanical properties of PAA-CMC-TDM can be regulated, but the functional activity of TDM remains unaffected. Cytological studies and animal models of hard tissue defects show that the hydrogel can promote the odontogenesis or osteogenic differentiation of mesenchymal stem cells, adapt to irregular hard tissue defects, and promote in situ regeneration of defective tooth and bone tissues. In summary, this paper shows that the injectable TDM hydrogel based on biomimetic mineralization theory can induce hard tissue formation and promote dentin/bone regeneration.

## Introduction

Biological hard tissues, such as teeth, vertebrate bones, and mollusk shells, are organic–inorganic composite functional materials with excellent mechanical properties; thus, they play important physiological roles in an organism while constantly renewing and repairing themselves^1,2^. Meanwhile, owing to factors such as infection, trauma, tumor-caused large areas of bone defects and insufficient blood supply, normal self-repair is difficult, especially in the case of maxillofacial hard tissue^3,4^. Maxillofacial tissue engineering based on structural and functional reconstruction is beneficial to the physiological repair of maxillofacial trauma. By simulating the biological tissue structure, a personalized tissue engineering repair module has been constructed to realize the functional repair and regeneration of the defect area, such as the repair of the mandibular defect, construction of bioengineered teeth, and regeneration of the pulp–dentin complex^5,6^. Ideally, for areas with insufficient blood supply or complex defects, an osteoinductive scaffold can be built using functional filling materials by preventing the filling of the dead space, mediating the immune activity and stem-cell homing and differentiation, and promoting the regeneration of restorative bone or dentin structure to achieve functional preservation or restoration^7^.

The biomaterials commonly used for hard tissue repair and regeneration are divided into four main categories: biogenic bone or bone derivative, bio-ceramic-based materials, synthetic or natural polymer materials; and double or multiple composite biological bone repair materials^8–10^. However, the physiological repair of bone or dentin restoration using simple calcium mineral materials is insufficient. Traditional autologous or allogeneic, heterogeneous bone materials exhibit high bioactivity and bone inducibility. While their sources, ethical issues, immunogenicity, and other potential risks cannot be underestimated^11^.

The dentin matrix, which contains collagen proteins and many non-collagen proteins (NCPs) associated with mineralization, is a type of autologous acquisition biomineral material^12–15^. It plays a significant role in the osteogenesis and odontogenic lineage differentiation of mesenchymal cells and can be used for repairing hard tissue defects^16–19^. Based on tissue regeneration and repair requirements, clinical bioremediation materials are expected to be nontoxic and nonirritating and exhibit stable properties, good biocompatibility, sustainable efficacy, and low technical sensitivity^20–22^. However, due to the highly mineralized structure and particle conditions of the treated dentin matrix (TDM), it is difficult to be reconstructed rapidly after the destruction of covalent bond scaffolds, and its functional activity in vivo is unstable in the absence of adhesive carriers^23^.

Unlike the structure formed by covalent bonds, the dynamic bonding properties of noncovalent bonds, including ion interactions, hydrophobic interaction, metal coordination bonds, and hydrogen bonds, can impart self-healing and injectable properties to the materials^24^. In teeth, bones, and other biominerals, NCPs and proteoglycan, as binders of mineralized collagen and stabilizers of the amorphous calcium phosphate (ACP) precursor, form the basis for the unique mechanical properties of hard tissue with resistant elastic binding^25–27^. Inspired by these structural features, the building of hard tissue repair materials with suitable mechanical properties and efficient healing properties has been recently explored by mimicking the mineral phases of natural biological bone in terms of its chemical composition and structural features^28–31^.

However, natural or recombinant NCPs are expensive and clinically less practical. Several studies have used polymers to mimic NCP analogs to construct composites that bind inorganic minerals and organic polymers^32–34^. For example, polyacrylic acid (PAA) is a highly hydrophilic synthetic polymer with a rich carboxyl group that can be transformed into a hydrogel through crosslinking. Rigid hybrid materials similar to mineral plastics with high plasticity and self-healing ability can be prepared by combining PAA with *Ca*^*2+*^ and *Mg*^2+ ^^28,35,36^. The assembly of nano-hydroxyapatite (nHA), sodium carbonate, and PAA through supramolecular assembly constructs biomineralized hydrogels with osteoinductive potential^30^.

Chitosan is a natural cationic polysaccharide polymer extracted from a chitosan shell. A soluble carboxylated chitosan (CMC) is obtained from the carboxylation reaction. Chitosan and its derivatives are widely used in hydrogels, healing trauma-class biomaterials, and tissue-engineered matrix materials because of their excellent polyelectrolyte action, biodegradability, biological activity, and chemical functional flexibility^37–39^. Based on the principle of biomimetic mineralization, PAA and CMC are often reacted to form copolymers or used to prepare hybrid hydroxyapatite (HA)^40–42^.

**Fig. 1:**
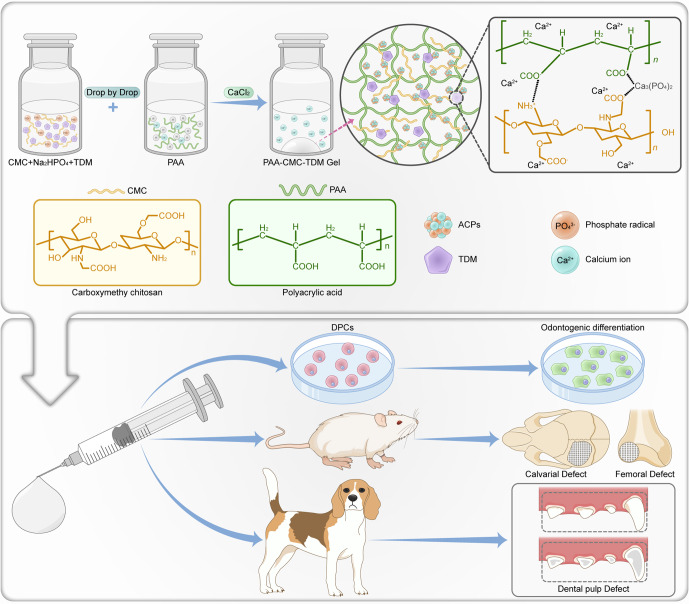
Schematic diagram of PAA-CMC-TDM mineralized hydrogel for hard tissue regeneration and the experimental design. The mineralized particles such as TDM or nHA are uniformly dispersed in the CMC/Na_2_HPO_4_ solution and mixed with the PAA solution by dripping. After the components are fully mixed, saturated calcium chloride is added to promote the formation of the hydrogel. The ability of bone regeneration was evaluated by cytological experiments in vitro, repair experiments of the femur and calvarial defects in rats and dental pulp defect model experiments in Beagle dogs.

As shown in Fig. 1 from the perspective of biomimetic mineralization, based on supramolecular self‐assembly of PAA, CMC, and *Ca*^*2+*^, this study constructs PAA-CMC-TDM, where TDM acts as the mineralization core template. The *Ca*^*2+*^*·COO*^*−*^ coordination bond in PAA-CMC-TDM stabilizes TDM and forms a dynamic noncovalent bond scaffold with hydrogen and ionic bonds simultaneously, which imparts the hydrogel-suitable self-healing and shear-thinning properties. The hydrophilicity of PAA-CMC-TDM is conducive to the release of its functional active components in the physiological environment, thus inducing the odontogenic and osteogenic differentiation of mesenchymal stem cells, and ultimately promoting the in situ regeneration of various defects in hard tissues, such as bone and teeth.

## Results

### Preparation of mineralized hydrogel

**Fig. 2:**
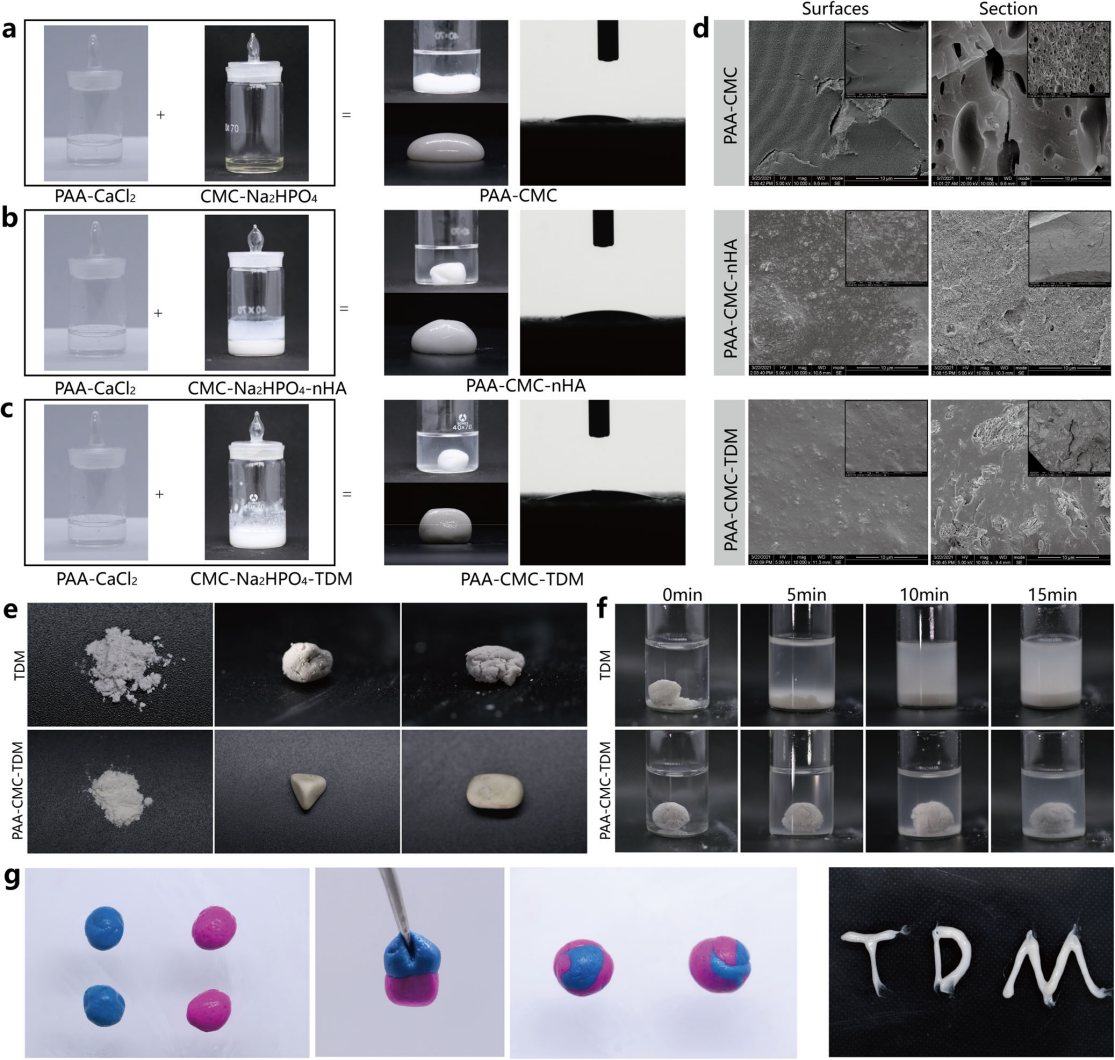
Preparation and morphological characterization of materials. **a**–**c** Preparation process and hydrophilic evaluation of the PAA-CMC, PAA-CMC-nHA and PAA-CMC-TDM. The hydrogels showed favorable hydrophilia with a relatively low water contact angle. **d** The SEM surface and section observation of freeze-dried hydrogels. **e** TDM and PAA-CMC-TDM powder were mixed with water and molded, respectively, PAA-CMC-TDM powder could be formed smoothly and its shape was relatively complete. **f** Images of PAA-CMC-TDM hydrogel and TDM clumps sonicated for 0–15 min. **g** Self-healing process of PAA-CMC-TDM. The hydrogels were cut into two pieces and then reassembled at room temperature to form a completely new one. Hydrogel can be extruded through a syringe. Scale bar (**d**): 10 and 100 µm.

To improve the formability of TDM powder and expand its application range, we designed a TDM carrier with PAA as the NCP simulant and CMC as the glycosaminoglycan simulant, promoted the polymerization of saturated *Ca*^*2+*^, and constructed a plastic and injectable noncovalent mineral hydrogel (Fig. 1). As shown in Fig. 2a–c, mineralized particles, such as TDM or nHA, were uniformly dispersed in the CMC/Na_2_HPO_4_ solution and mixed with the PAA solution through dripping. The PAA-CMC (Fig. 2a), PAA-CMC-TDM (Fig. 2b), and PAA-CMC-nHA (Fig. 2c) hydrogels formed were milky white, insoluble in water, and hydrophilic. This strategy does not require a complex molecular design or sophisticated synthesis process^30^. Furthermore, under 500 μL of PAA solution and 0.1 g of TDM, the mass and volume of the hydrogels can be stabilized by adjusting the pH value, the concentration of CaCl_2_ and Na_2_HPO_4_, and the molecular weight and content of CMC within a certain range (Supplementary Fig. 2). Based on these data and considering that the material needs to be used in the physiological environment, we determined that the system must be prepared with the material having a pH of 7.0, less than or equal to 15 W molecular weight of CMC, Na_2_HPO_4_ concentration of 0.25 mol/L (5 mL), and CaCl_2_ concentration of 1 mol/L (2 mL).

The scanning electron microscopy (SEM) results showed that the PAA-CMC surface was complete, but holes of different sizes were visible at the cross-section, suggesting a connected micropore structure inside (Fig. 2d). Zhao et al.^30^ suggested that the mineralized hydrogel exhibited an interconnected microporous structure ranging from 100 to 1000 nm. However, the surface and cross-section of PAA-CMC-TDM were rough, and TDM particles with dentine tubules were visibly exposed, accompanied by an irregular protrusion. However, the pore structure was not evident. According to the STEM-EDX results presented in Supplementary Fig. 3, *Ca*^*2+*^ was widely and uniformly distributed in the hydrogels. Among them, the concentration of *Ca* in PAA-CMC-ACP was 89%, which is higher than those in PAA-CMC-nHA (65%) and PAA-CMC-TDM (78%). In addition, the content of *P* in PAA-CMC-nHA was 35%, which is higher than those in PAA-CMC-TDM (22%) and PAA-CMC-ACP (11%).

The PAA-CMC-TDM hydrogels described were injected through a 1-mm needle without clogging (Fig. 2g). To evaluate their self-healing ability, the hydrogels were stained with blue and red dyes. They were cut into two pieces each and then reassembled to form completely integrated gels at room temperature. Moreover, it can be seen from the SEM results that the fusion can be observed on the cross-section after the materials are in contact with each other (Fig. 2g and Supplementary Fig. 4). After being subjected to 15 min of ultrasonic shock, the TDM material disintegrated rapidly in water. In contrast, the formed PAA-CMC-TDM disintegrated only slightly, maintaining a stable shape of the material (Fig. 2f). In addition, compared to TDM powder, PAA-CMC-TDM exhibited significantly improved plasticity and could be reshaped differently to meet the requirements of different application scenarios (Fig. 2e).

### Stability of mineralized hydrogel

**Fig. 3:**
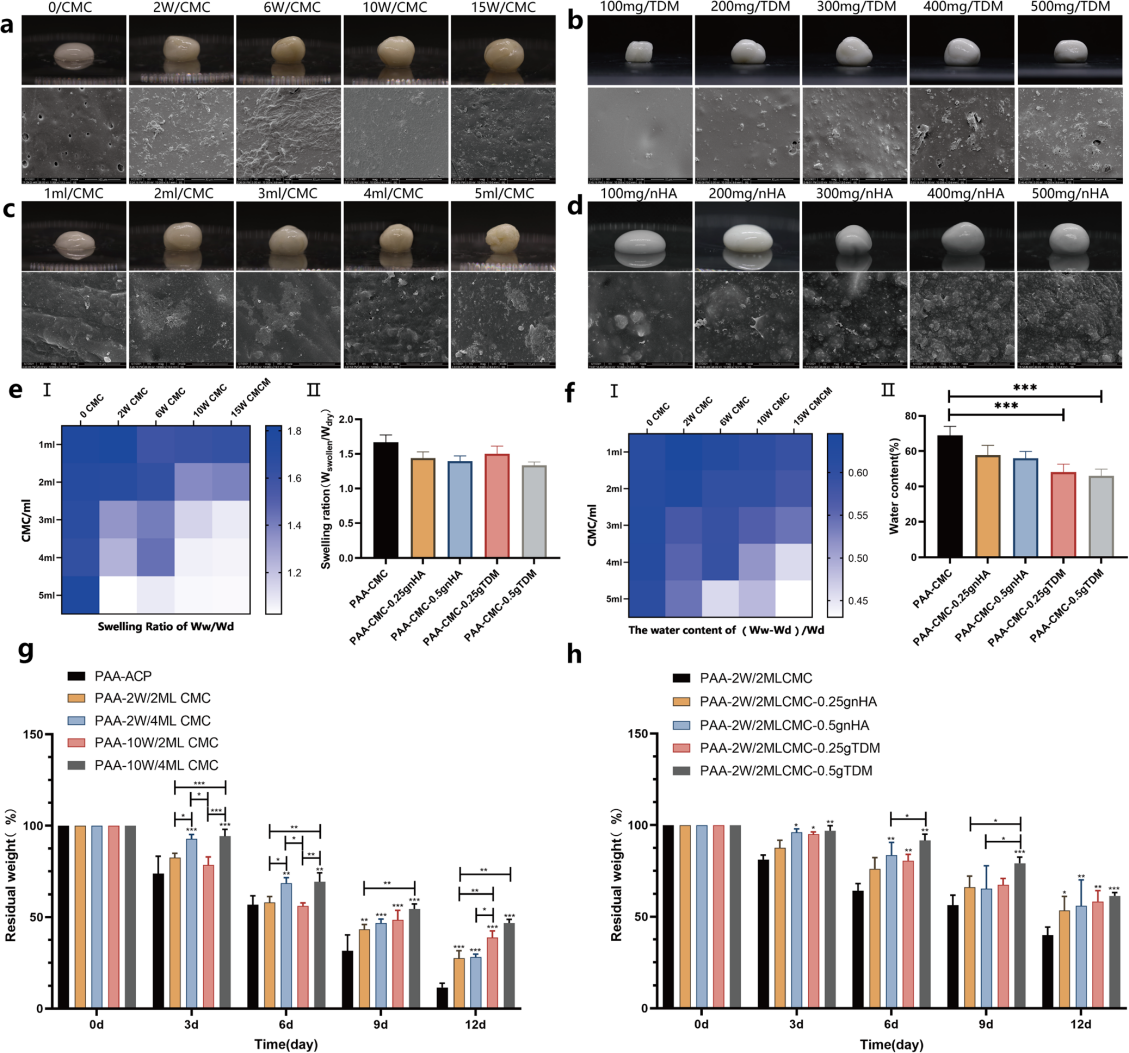
Study of the properties, swelling, and degradation of hydrogels after adjusting the material composition. **a** The optical images and SEM surface observation of hydrogels of identical content CMC with different molecular weights. **b** The optical images and SEM surface observation of hydrogels of identical molecular weights CMC with different content. **c** The optical images and SEM surface observation of hydrogels of TDM with different content. **d** The optical images and SEM surface observation of hydrogels of nHA with different content. **e** I. Swelling rate of hydrogels containing different molecular weights and contents of CMC at the same amount of TDM. II. Swelling rate of the hydrogels containing different amounts of TDM or nHA with the same amount of CMC. **f** I. The water content of hydrogels containing different molecular weights and contents of CMC at the same amount of TDM. II. The water content of the hydrogels containing different amounts of TDM or nHA with the same amount of CMC. **g** Residual weight of hydrogels containing different molecular weights and contents of CMC in PBS with 2 U/mL collagenase at 37 °C for 12 days. **h** Residual weight of hydrogels containing different amounts of TDM or nHA in PBS with 2 U/mL collagenase at 37 °C for 12 days. Error bars indicate standard deviation (*n* = 3); *p < 0.05, **p < 0.01, and ***p < 0.001 (two-way ANOVA). Scale bar (**a**–**d**): SEM, 10 µm.

PAA-CMC-TDM hydrogel has good maneuverability, but its stability may be affected by the external environment. It is therefore necessary to optimize the mechanical properties of hydrogels by adjusting their content and molecular weight, stability and swelling coefficient, and degradation ability. As shown in Fig. 3a, b, when CMC was added, the hydrogel’s fluidity and plasticity decreased and its molecular weight increased. In addition, PAA-CMC was formed stably and would not collapse due to gravity. The results of the SEM analysis revealed that the CMC changes hardly affected the microstructure; only the number of pores was reduced, and irregular nodules were formed. With the other conditions maintained the same, through the addition of mineral matrices such as TDM and nHA, the color of the hydrogel changed to milky white and the fluidity decreased. However, the hydrogel could be appropriately formed, and the stability was improved. Moreover, the filler exposure rates on the gel surface increased with the size of the mineral matrix, as observed in Fig. 3c, d.

Figure 3e, f shows that the increase in the molecular weight and volume of the added CMC reduced the material’s swelling rate, which in turn decreased its water content. There was no apparent effect of TDM or nHA on the swelling coefficient of the gel when other parameters were known. Mineral particles can reduce the water content of the hydrogel to some extent, and TDM is slightly more effective than nHA at reducing the water content. The degradation experiment of PAA-CMC-TDM lasted for 12 days (Fig. 3g, h). As the soaking time was increased, PAA-CMC series hydrogels exhibited varying degrees of degradation. In particular, CMC with high molecular weight and content could enhance the hydrogel stability and prolong the degradation time. Mineral matrix additions such as TDM/nHA also improve the hydrogel stability, but the type and quality of the mineral matrix has little effect on degradation.

### Physicochemical characterization of mineralized hydrogel

**Fig. 4:**
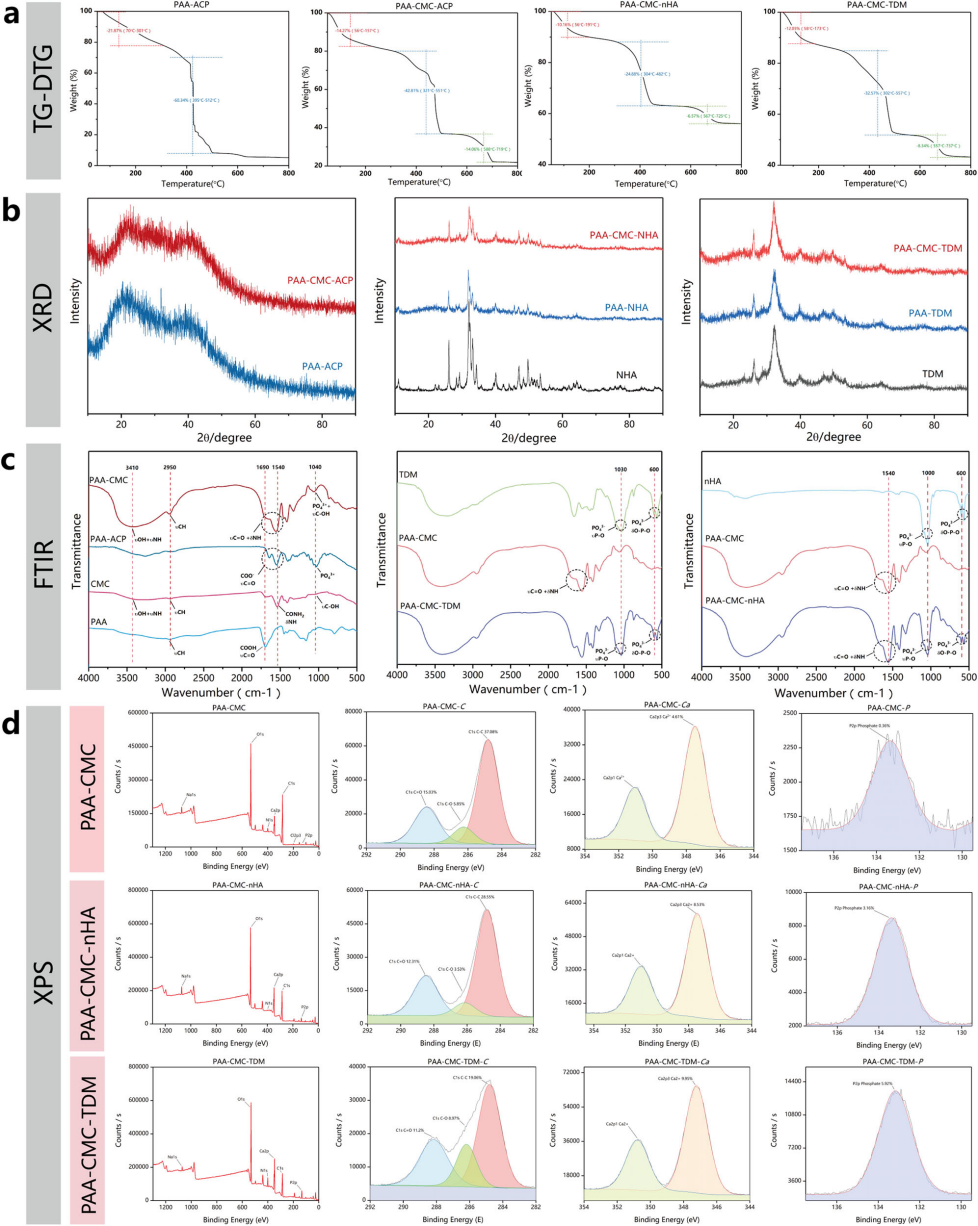
Components and structure characterization of hydrogels. **a** The TGA-DTG analysis curve of hydrogels PAA-ACP, PAA-CMC, PAA-CMC-nHA and PAA-CMC-TDM with temperature ranges from 50 to 800 °C. **b** The XRD patterns of the hydrogels and the mineral matrix fillers TDM and nHA. **c** ATR-FTIR spectra of PAA-ACP, PAA-CMC-ACP, PAA-CMC-nHA, PAA-CMC-TDM, CMC and PAA. **d** XPS spectrum and the high-resolution XPS spectra of *Ca 2p*, *C 1s* and *P 1s* of the fabricated PAA-CMC, PAA-CMC-nHA and PAA-CMC-TDM.

To determine the thermal properties of the mineralized hydrogels, the thermogravimetric-difference thermogravimetry analysis (TG-DTG) analysis was performed. Figure 4a shows the TGA curves of PAA-ACP, PAA-CMC-ACP, PAA-CMC-nHA, and PAA-CMC-TDM under airflow. As the temperature increased, water loss, PAA degradation, and CMC degradation occurred sequentially in these four gels. According to the TGA curves, for PAA-ACP, the weight ratios of calcium phosphate, PAA, and water were 20, 60, and 20 wt%, respectively, which is in line with previously obtained results^36^. In addition, compared to PAA-ACP, the quality of CMC-containing hydrogels decreased significantly at 600 °C. In relevant studies^43,44^, the water loss temperature of CMC was found to be approximately 80 °C. The decomposition temperature of the main chain fracture was approximately 260 °C, which released small molecular fragments; and the decomposition temperature was approximately 630 °C.

According to the mass of the last remaining residues in TGA, the contents of PAA-CMC-TDM and PAA-CMC-nHA were more than 40%, most of which were expected to be HA with a degradation temperature above 1000 °C. Under the same preparation conditions and the same mass of TDM and nHA, the PAA-CMC-TDM residue was lower than the PAA-CMC-nHA residue, which may be due to the collagen and functional proteins present in TDM. It has been reported that nonmineral components in dentin account for approximately 20% of the total mass^45^.

Furthermore, the crystal structures of the mineralized hydrogel characterized by X-ray diffraction (XRD) spectroscopy are presented in Fig. 4b. As shown in Fig. 4b, the XRD patterns of PAA-ACP and PAA-CMC-ACP were mound-like, indicating the amorphous characteristics of the hydrogels. The XRD profiles of the mineralized hydrogels supplemented with TDM and nHA were largely similar to that of the added mineral matrix. The diffraction peaks of nHA and TDM at 2*θ* were 25.7°, 28.6°, 31.5°, 34.2°, 40.1°, 47.1°, 49.8°, and 53.2°, which are consistent with the values specified in the XRD standard card of HA (JCPDS: 72–1243)^46^.

Meanwhile, the FTIR spectrum was drawn to identify the chemical structure of the resultant gels (Fig. 4c). In PAA-CMC, the 3400 cm^−1^ wide peak corresponded to the *υOH* and the *N-H* stretching vibration (*υNH*) derived from the CMC. In contrast to the PAA atlas, the peaks of *υC*=*O* (carboxylic acid carbonyl stretching vibration) in PAA-ACP and PAA-CMC decreased from 1690 to 1540 cm^−1^. Meanwhile, in PAA-CMC, the peak at 1550 cm^−1^ corresponded to the *N-H* in-plane bending vibrations (*δNH*), while that at 1320 cm^−1^ corresponded to the *C-N* stretching vibrations (*υCN*), both of which were the main characteristic peaks of the amide groups. In PAA-ACP and PAA-CMC, the peak at 1030 cm^−1^ was assigned to the stretching vibration of *PO*_*4*_^*3-*^ (*υPO*). In the infrared spectra of PAA-CMC-TDM and PAA-CMC-nHA, the peaks at 600 and 565 cm^−1^ corresponded to the in-plane bending vibration absorption of *O-P-O* (*δO-P-O*).

In addition, we used X-ray photoelectron spectrometer (XPS) to reveal the chemical composition and status of the mineralized hydrogels (Fig. 4d). The results revealed that these hydrogels contained *Na*, *O*, *N*, *Ca*, *C*, *Cl*, and *P* elements. The *Ca* and *P* elements present in PAA-CMC were mainly derived from calcium phosphate, while those in PAA-CMC-TDM and PAA-CMC-nHA were mainly derived from the HA component. In PAA-CMC and PAA-CMC-nHA, the binding energies of *C 1s* peaks at 284.8, 286, and 288 eV were assigned to *C*=*O*, *C-O*, and *C-C* from PAA and CMC in hydrogels, while some binding energy of *C 1s* in PAA-CMC-TDM might have resulted from the collagen and NCPs in the dentin matrix.

### Rheological characterization of mineralized hydrogel

The rheological and viscoelastic properties of the mineralized hydrogel were investigated through rheological studies. Here, the elasticity and fluidity indicators of the materials were analyzed in terms of the storage modulus (*G’*) and loss modulus (*G”*)^47^. According to the oscillatory rheological characterization, the molecular weight and content of CMC and the mineral matrix fillers (TDM and nHA) affected the mechanical properties of the hydrogels (Supplementary Fig. 5). The addition of a few mineral particles can decrease the *G*’ and *G*” values of the gel to a certain extent. However, with the increasing number of particles added to the material (nHA >0.1 g, TDM >0.2 g), the mechanical properties of the whole hydrogel will gradually improve (Supplementary Fig. 5a, b). In addition, the value of *G*’ increases with the molecular weight of CMC, which indicates an increase in the material hardness. With the increase in the amount of CMC with the same molecular weight, both *G*’ and *G*” increased at first and then decreased (Supplementary Fig. 5c–f). Considering that the filling material for irregular hard tissue defects needs to balance the mobility and stability of the gel, hydrogels containing CMC (20,000 MW, 4 mL, 5%), TDM (0.5 g)/nNA (0.5 g), and 35% PAA (1 mL) were compared and referenced for further study.

**Fig. 5:**
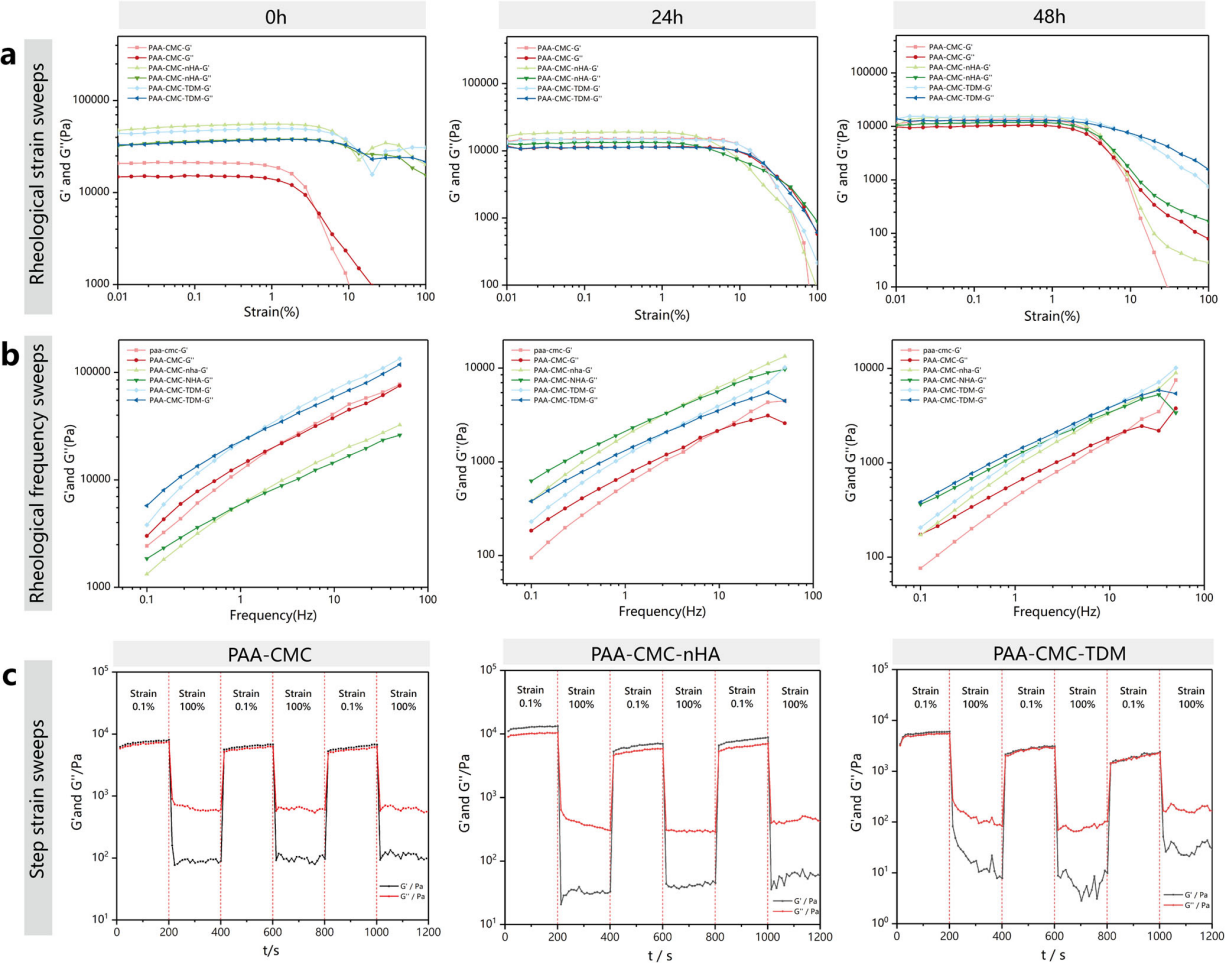
Rheological characterization of the mineralized hydrogel. **a** Rheological strain sweeps of the hydrogel (strain = 0.01–100%, temperature: 20 °C, and frequency: 10 Hz). **b** Rheological frequency sweeps of the hydrogel (temperature: 20 °C and stress: 10 Pa, 0.1–50 Hz). **c** Step–strain sweeps of the hydrogel (temperature: 20 °C and frequency: 10 Hz, strain: 0.1%, 200 s, 100%, 200 s).

Based on the appellate components, we prepared PAA-CMC, PAA-CMC-nHA and PAA-CMC-TDM and assessed the changes in their mechanical properties after 24 and 48 h of immersion in phosphate-buffered saline (PBS). Stress sweeps (10 Hz) were first performed to obtain the hydrogels’ linear viscoelastic range (LVR), as shown in Fig. 5a. The LVR of the hydrogels with TDM/nHA was slightly wider than that of PAA-CMC before immersion, while the LVR of PAA-CMC widened after 24 h of immersion in PBS. After 48 h of PBS immersion, no significant differences were observed in the LVR of the three hydrogels.

Then, frequency sweep tests were conducted within the LVR of the hydrogels. As shown in Fig. 5b, with an increase in the vibration frequency, the *G’* and *G”* values of the three types of hydrogels increased. At the initial stage, the value of *G”* was higher than that of *G’*, and the hydrogels behaved like viscous materials. When the frequency reached a certain value, the *G’* value increased beyond the *G”* value. Research has shown that hydrogels behave as viscoelastic materials^47,48^. After being soaked in PBS, their *G’* and *G”* values decreased. A higher vibration frequency was required when the *G’* value exceeded the *G”* value, indicating that the swollen hydrogels behaved like a viscous material lacking elastic characteristics^49^.

Next, step–strain measurements for strains ranging from 0.1 to 100% were performed to evaluate the repeated self-healing property of the hydrogels, following hydrogel rupture by a mechanical shearing force (Fig. 5c)^29,50^. With a small-amplitude oscillatory shear (strain = 0.1%) applied to all three hydrogels, their *G’* values increased slightly beyond their *G”* values. Then, under a large-amplitude oscillatory shear lasting for 200 s (strain = 100%), the *G”* values of all hydrogels increased beyond the *G’* values, indicating fluid-like behavior and failure of the hydrogels; however, the values immediately returned to the original solid-like behavior after the shear was reduced. This property was also maintained in several repeatable cycles and remained largely unaffected by the addition of mineral particles.

### In vitro effects on cell biological behavior of mineralized hydrogel

Dental pulp mesenchymal stem cells (DPCs) with high proliferation potential and multi-lineage differentiation ability can be obtained from the pulp tissue of young permanent teeth and deciduous teeth, which exhibit osteogenic, chondrogenic, adipogenic, and neurogenic potential^51^. Related studies have shown that DPCs can be used as the seed cell for tooth and bone tissue engineering and might be suitable for evaluating the osteogenesis and odontogenesis induction abilities of the materials^52,53^. We obtained spindle-shaped DPCs that maintained their morphology after passage three, and these cells expressed a mesenchymal marker Vimentin (Supplementary Fig. 6a). Transwell assay and scratch experiment were performed to explore the ability of DPCs to migrate both vertically and horizontally (Supplementary Fig. 6b–e).

According to the statistical results obtained for the three independent experiments shown in Supplementary Fig. 6d, e, the extracts of PAA-CMC-nHA showed an inhibitory effect on cell migration compared to the control group, while the conditional media of CMC did not contribute to cell migration. The promoting effect of the extracts of PAA-CMC-TDM and TDM on the cells was not significantly different from that of the control group. The results of the proliferation of human dental pulp stem cell (hDPC) analysis with Cell Counting Kit-8 (CCK-8) are presented in Supplementary Fig. 6f. In addition, PAA-CMC-nHA exhibited a slight inhibitory effect on cell proliferation. In contrast, the extracts of PAA-CMC-TDM and TDM had no evident effect on cell proliferation.

**Fig. 6:**
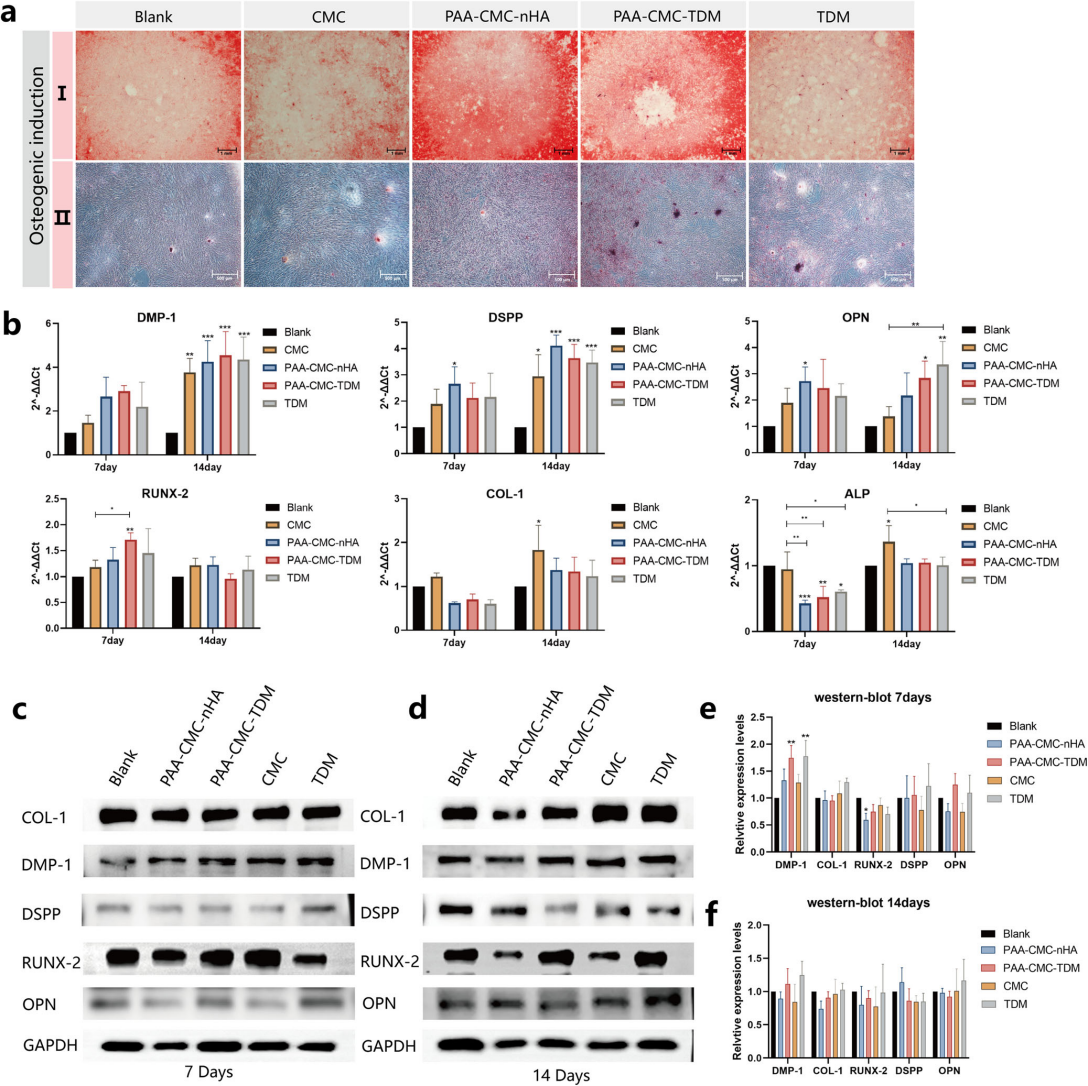
Effect of hydrogel on odontogenic differentiation of DPCs. **a** Alizarin Red S staining of DPCs cultured in the control group (PBS), TDM, CMC and hydrogel groups. Red indicates mineral content. **b** RT-PCR of odontogenic differentiation marker genes DSPP, DMP-1, ALP, COL-I, OPN, and RUNX-2 after treatment with TDM, CMC and mineralized hydrogels for 7 days and 14 days. **c**, **d** Western blot analyses of odontogenic differentiation marker DSPP, DMP-1, ALP, COL-I, OPN, and RUNX-2 after treatment with TDM, CMC and mineralized hydrogels for 7 days (**c**) and 14 days (**d**). **e** Gray-scale analysis of western blot analysis results for 7 days. **f** Gray-scale analysis of western blot analysis results for 14 days. Error bars indicate standard deviation (*n* = 3); *p < 0.05, **p < 0.01, and ***p < 0.001 (two-way ANOVA). Scale bar (**a-I**):1 mm; (**a-II**): 500 µm.

Then, we evaluated the ability of the hydrogels to promote dental osteogenic differentiation through osteogenic induction culture and alizarin red S (ARS) staining (Fig. 6a). The results showed that, compared to the control group and CMC group, ARS staining in PAA-CMC-nHA, PAA-CMC-TDM, and TDM groups was deeper. And there were evident calcium nodules that could be observed in PAA-CMC-TDM and TDM groups. Meanwhile, through RT-PCR and western blot analyses, we detected the changes in related indexes of hard tissue differentiation at protein and RNA levels after 7 and 14 days of induction for the cells. As detailed in Fig. 6b, compared to the control group, with PAA-CMC-nHA, PAA-CMC-TDM, and TDM induction for 14 days, the RNA expression levels of osteopontin (OPN), dentin matrix protein-1 (DMP-1), and dentin sialophosphoprotein (DSPP) were significantly enhanced in hDPCs. The changes in COL-I in the mineralized hydrogels and TDM were not evident (*p* > 0.05) during the 14-day induction. In contrast, for the 7-day induction, the PAA-CMC-TDM extract upregulated the RUNX-2 (*p* < 0.05) expression of hDPCs compared to the control group. Meanwhile, the extract of the mineralized hydrogels PAA-CMC-nHA, PAA-CMC-TDM, and TDM significantly decreased the alkaline phosphatase (ALP) gene expression under 7-day induction. In contrast, CMC induced a mild upregulation of ALP and COL-I expression levels under 14-day induction. The results of the western blot analysis presented in Fig. 6c–f revealed that, under 7-day induction, TDM and PAA-CMC-TDM improved the expression levels of DMP-1, DSPP, and OPN proteins compared to the control group. At the same time, compared to the control group, the expression of RUNX-2 in the other four groups decreased after being induced for 7 and 14 days, especially in PAA-CMC-nHA. In addition, the COL-I expression increased slightly after 7 days of TDM and CMC induction. However, under 14-day induction, differences were not apparent across groups except for the decreased expression in the PAA-CMC-nHA group.

### In vivo biocompatibility of mineralized hydrogels

**Fig. 7:**
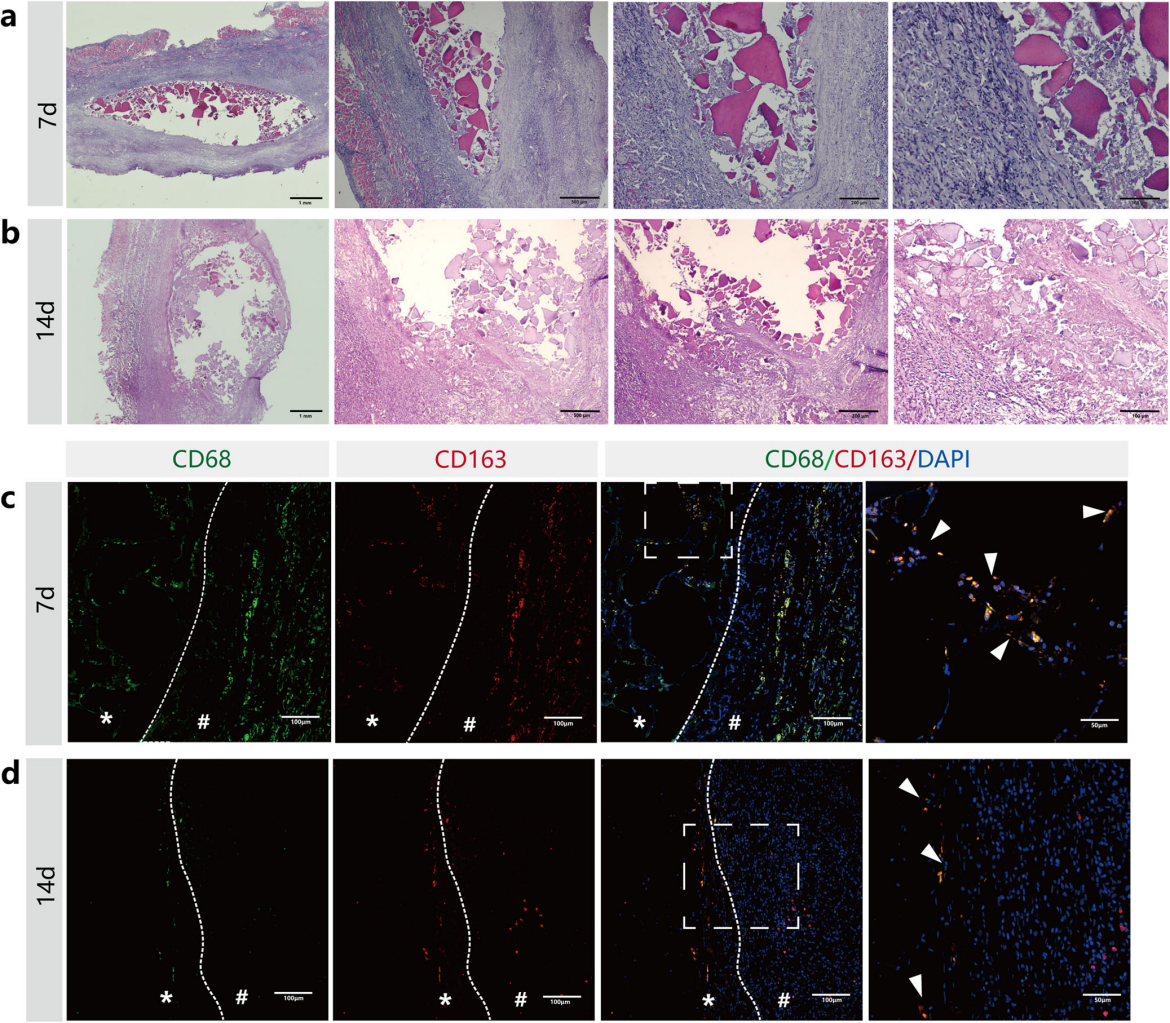
HE staining and fluorescent immunohistological staining of PAA-CMC-TDM implanted subcutaneously in rats. **a**, **b** H&E staining histologic sections of skin tissues surrounding PAA-CMC-TDM for 7 days (**a**) and 14 days (**b**). **c**, **d** Fluorescent immunohistological staining histologic sections of CD68 (Green) and CD163 (Red) of skin tissues surrounding PAA-CMC-TDM for 7 days (**c**) and 14 days (**d**). *The material. ^#^The surrounding tissue. Scale bar (**a**): 1 mm, 500 µm, 200 µm, 100 µm; (**c**, **d**, CD68): 100 µm; (**c**, **d**, CD163): 100 µm; (**c**, **d**, CD68/CD163/DAPI): 100 and 50 µm.

PAA-CMC-TDM was implanted subcutaneously in the rats to directly evaluate its degradation and inflammatory reaction. As shown in Fig. 7a, b, under H&E staining, after 7 days, the hyperplasia of fibrous connective tissue around the subcutaneous specimen wrapped the remaining material, and undispersed TDM particles were visible in the material area, while the scaffold components of PAA and CMC remained invisible. After 14 days of implantation, large TDM particles could still be observed in the implantation area. Most of the small mineral particles were wrapped in fibrous connective tissue and decomposed gradually.

In addition, fluorescent immunohistological staining of the macrophage marker CD68 and M2 macrophage marker CD163 was used to characterize the local immune response (Fig. 7c, d)^54^. CD68+ and CD163+ macrophage invasion at the interface between PAA-CMC-TDM and the surrounding tissue was observed on day 7. However, the CD68 and CD163 expression levels decreased after 14 days, which indicated that the subcutaneous tissue had a mild and early inflammatory reaction with the mineralized hydrogels, with concomitant tissue healing and regeneration.

### In vivo bone tissue regenerative properties of mineralized hydrogel

**Fig. 8:**
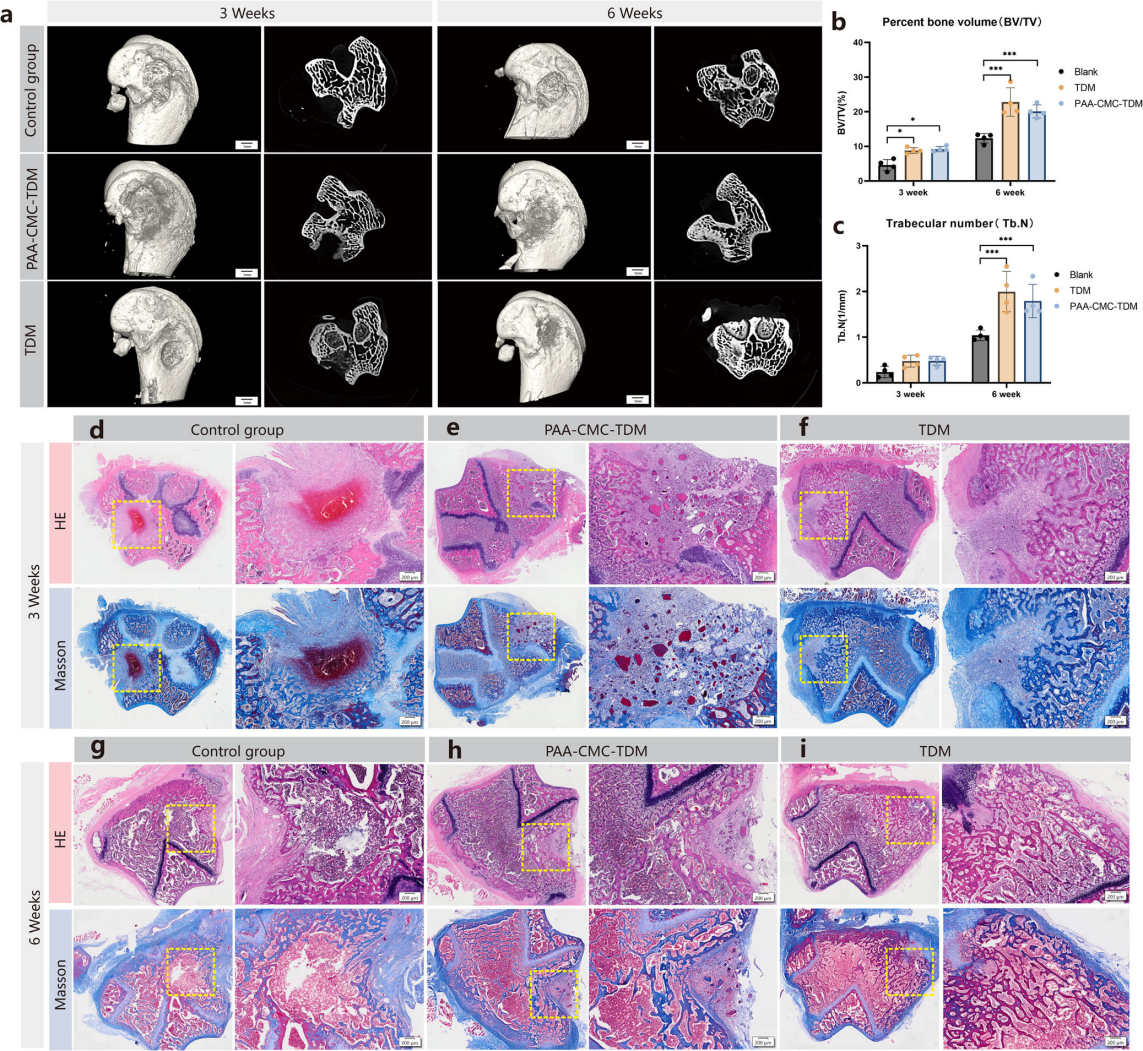
In vivo bone regeneration on femoral defect after 3 and 6 weeks of implantation of mineralized hydrogels. **a** Micro-CT assessment of bone regeneration in femoral defect at 3 and 6 weeks after implantation and 3D micro-CT reconstruction was carried out to observe the bone microstructure. **b** The quantitative analysis of bone volume/tissue volume (BV/TV) ratio by micro-CT. **c** The quantitative analysis of trabecular number (Tb.N, 1/mm) by micro-CT. **d**–**f** The HE staining and Masson’s trichrome staining evaluation of bone regeneration in defects at 3 weeks after implantation. **g**–**i** The HE staining and Masson’s trichrome staining evaluation of bone regeneration in defects at 6 weeks after implantation (Control group, PAA-CMC-TDM group, TDM group). Error bars indicate standard deviation (*n* = 4); *p < 0.05, **p < 0.01, and ***p < 0.001 (two-way ANOVA). Scale bar (**a**): 1 mm; (**d–i**): 200 μm.

**Fig. 9:**
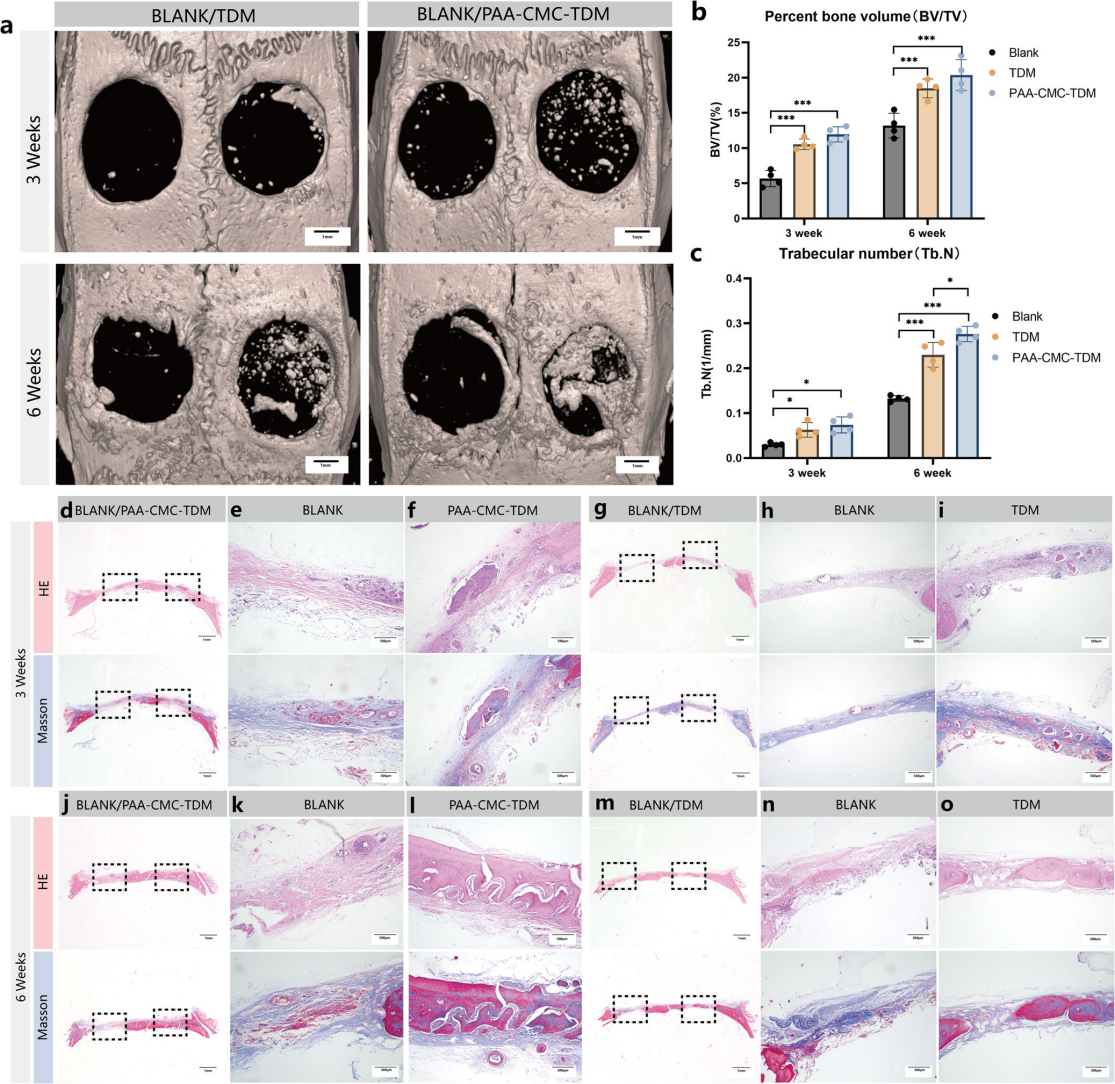
In vivo bone regeneration on calvarial defect after 3 and 6 weeks of implantation of mineralized hydrogels. **a** Micro-CT assessment of bone regeneration in calvarial defect at 3 and 6 weeks after implantation, and 3D micro-CT reconstruction was carried out to observe the bone microstructure. **b** The quantitative analysis of bone volume/tissue volume (BV/TV) ratio by micro-CT. **c** The quantitative analysis of trabecular number (Tb.N, 1/mm) by micro-CT. **d**–**i** The HE staining and Masson’s trichrome staining evaluation of bone regeneration in calvarial defect at 3 weeks after implantation of PAA-CMC-TDM and TDM. **j**–**o** The HE staining and Masson’s trichrome staining evaluation of bone regeneration in calvarial defect at 6 weeks after implantation of PAA-CMC-TDM and TDM. Error bars indicate standard deviation (*n* = 4); *p < 0.05, **p < 0.01, and ***p < 0.001 (two-way ANOVA). Scale bar (**a**): 1 mm; (**d**, **j**, **g**, **m**): 1 mm; (**e**, **f**, **h**, **I**, **k**, **l**, **n**, **o**): 500 μm.

The cranial bone and femur defect models were used to evaluate the effects of PAA-CMC-TDM and TDM on bone defect repair. As shown in Fig. 8a–c, in the femoral defect of the 6th week, compared to the blank control group, evident bone matrix filling could be observed in the bone defect area of the TDM and PAA-CMC-TDM groups. Through BV/TV and trabecular number (Tb.N) analyses, the amount of new bone regeneration and bone trabeculae in the TDM group were slightly higher than those in the PAA-CMC-TDM group. The numbers of new bone trabeculae and bone formation in the TDM and PAA-CMC-TDM groups were higher than those in the control group. According to the calvarial defect results shown in Fig. 9a–c, there was only a little new bone formation in the control group, while the calvarial defect was significantly reduced in the TDM and PAA-CMC-TDM groups. Furthermore, the bone formation and Tb.N in the PAA-CMC-TDM group were slightly higher than those in the TDM group.

To further observe the growth and repair of bone at the bone defect, we stained the demineralized bone tissue sections with H&E and Massion staining. As observed in Figs. 8d–i and 9d–o, after 6 weeks, there was no evident inflammatory reaction in the histological sections of each group, and the tissue cells grew normally. In the femur and calvarial defect models, compared to the control group, significant bone healing occurred in PAA-CMC-TDM and TDM groups after 6 weeks of implantation. In the femoral defect, many coarse new bone trabeculae were found in the defect area of the TDM group after 6 weeks, while the collagen fiber tissue and few residual TDM particles could be seen in the lateral part of the defect in PAA-CMC-TDM in addition to the bone trabeculae and bone-like matrix (Fig. 8h, i). In the blank group, blood clots and repaired trabecular structures were observed in the defect area (Fig. 8d, g). In the calvarial defect, after 6 weeks, there was an evident formation of new bone and osteoid matrix in the defect area of the PAA-CMC-TDM group, and the defect healed (Fig. 9j–l). In the TDM group, the osteoid matrix and new bone tissue were also found in the periphery and center of the defect, and the bone defect was partially healed (Fig. 9m–o). The bone defect in the blank control group was still evident, and only a small amount of new bone matrix could be observed at the edge at 6 weeks (Fig. 9k, n).

To evaluate the biosafety of the materials in the bone repair process, the heart, liver and kidney of the defect rats in the experimental group were observed histologically, and no significant abnormalities were found in them compared to the healthy Sprague Dawley (SD) rats (Supplementary Fig. 8).

### Pulp capping material of mineralized hydrogel in the dog model

**Fig. 10:**
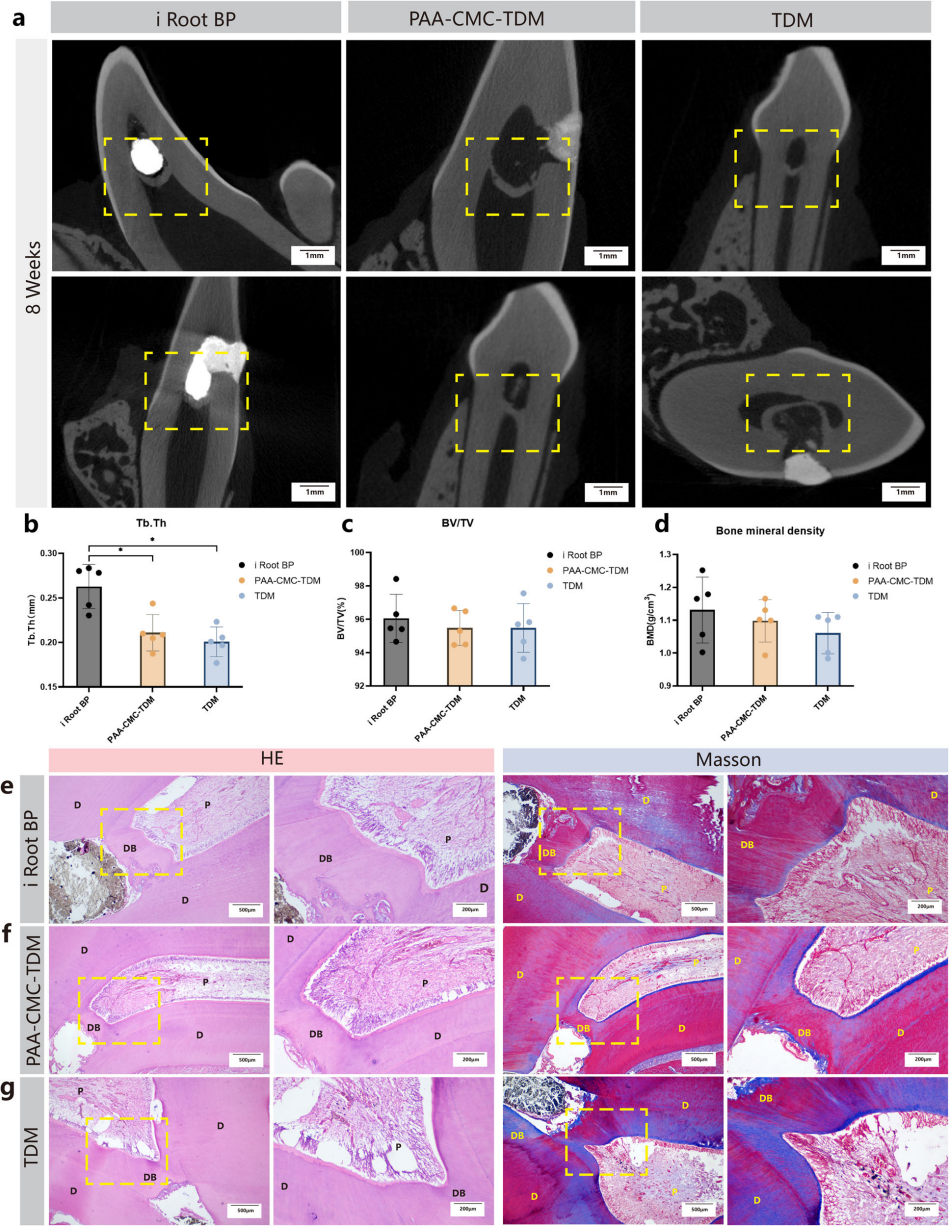
The mineralized hydrogels as pulp capping material, evaluated in vivo in Beagle dogs. **a** Micro-CT assessment of dentin bridge in dog tooth at 8 weeks after pulp capping with i Root BP plus, PAA-CMC-TDM and TDM. **b** The quantitative analysis of the thickness of the formed dentin bridge (Tb.Th) by micro-CT. **c** The quantitative analysis of dentin bridge mineralization ratio (BV/TV) by micro-CT. **d** The bone mineral density analysis of dentin bridge by micro-CT. **e** The HE staining and Masson’s trichrome staining evaluation of 8 weeks pulp capping with i Root BP plus in dog tooth. **f** The HE staining and Masson’s trichrome staining evaluation of 8 weeks pulp capping with PAA-CMC-TDM in dog tooth. **g** The HE staining and Masson’s trichrome staining evaluation of 8 weeks pulp capping with TDM in dog tooth. D dentin, P pulp tissue, DB dental bridge. Error bars indicate standard deviation (*n* = 5); *p < 0.05, **p < 0.01, and ***p < 0.001 (one-way ANOVA). Scale bar (**a**): 1 mm; (**e**, **f**, **g**, HE/Left): 500 μm; (**e**, **f**, **g**, HE/Right): 200 μm; (**e**, **f**, **g**, Masson/Left): 500 μm; (**e**, **f**, **g**, Masson/Right): 200 μm.

For evaluating the ability of PAA-CMC-TDM to promote reparative dentin formation and explore its possibility for vital pulp preservation, we compared the hard tissue induction abilities of TDM, PAA-CMC-TDM and i Root BP plus using the Beagle dog dental pulp defect model of 8 weeks (Supplementary Fig. 7). The micro-CT results (Fig. 10a) showed that the mineralized dentin layer was formed in the eight teeth in the i Root BP plus group (8/8), while dentin bridge formation was observed in five teeth in the PAA-CMC-TDM group (5/8) and five teeth in the TDM group (5/8). Through the quantitative analysis of the thickness and mineralization degree (BV/TV and bone mineral density) of the formed dentin bridge, the thickness and volume of the mineralized tissue in the i Root BP plus group were slightly larger than those in the TDM and PAA-CMC-TDM groups; however, there was no significant difference between the two groups (Fig. 10b). Meanwhile, the three groups showed no significant difference in the mineralization degree of the dentin bridge (Fig. 10c, d).

The results of the histological analysis showed that the pulp tissue of the experimental teeth with the dentin bridge was normal and there was no evident inflammatory reaction (Fig. 10e–g). The structure of dentin-like tubules could be observed in the reparative dentin of the TDM, PAA-CMC-TDM, and the i Root BP plus groups, similar to the naturally restored third-stage dentin. In all groups, polarized high columnar odontoblast-like cells were observed in the dental pulp tissue near the dentin bridge. In the i Root BP plus group (Fig. 10e), the complete dentin bridge was located on the pulp side of the material, and there was no gap, crack or pore between the dentine bridge and dentin tissue. Some areas of the dentine bridge showed bone-like dentin and the structure of dentinal tubules was disordered. Few calcified masses and hyperplastic blood vessels were observed in the dental pulp, but there was no evident increase in the fibrous connective tissue. In addition, the vascular content and fibrous connective tissue were significantly increased in the pulp tissue of the PAA-CMC-TDM group (Fig. 10f) and TDM group (Fig. 10g). In the TDM group, some TDM particles remained, and calcified spots were formed in the pulp on the pulpal side, while no evident free TDM particles and collapse of the capping pulp material were observed in the PAA-CMC-TDM group.

## Discussion

Mineralized tissue, such as bone and dentin has almost the same chemical composition. They are composed of 18–34% collagen, 1–2% NCPs, and 60–70% HA^1^. Dynamic reshaping is one of the characteristics of these hard tissues. After being damaged, tooth/bone can repair and regenerate itself through directional migration and differentiation of stem cells, dynamic balance of osteogenesis-osteoclast and secretion and mineralization of the hard tissue matrix. However, self-repairing of the defect tissue is difficult when the scope of the defect is too large or when the materials exchanged in the defect area are limited^7,55^. For the repair and regeneration of refractory hard tissues, the tissue engineering technique can be used to construct composite structures containing inducible scaffolds and hard tissue filling materials^7,11^.

The treated acellular bone matrix and dentin matrix can serve as a growth factor repository for hard tissue repair, such as bone morphogenetic proteins, transforming growth factor‑β, DMP-1 and OPN^1,56,57^. In addition, these mineral matrix materials have porous scaffolds that match their physiological functions, such as the osteon and dentinal tubule. Because of these, they exhibit osteoconduction and osteoinduction, making them ideal materials for hard tissue repair. The osteoconductive material can guide the growth of blood vessels and cells involved in bone regeneration and can participate in bone repair as a three-dimensional structure for hard tissue deposition^58,59^. In contrast, the osteoinductive material can induce undifferentiated mesenchymal stem cells to form bone progenitor cells and generate bone at ectopic sites^60,61^.

Dentin matrix materials exhibit better treatment characteristics and easier autologous acquisition, and are less immunogenic, making them attractive graft materials^62–65^. However, the granulated mineral matrix has no stable interaction force in the physiological environment, so it is difficult to maintain the shape and function for a long time, which is not conducive to application in an environment with irregular defects and complex structures^66^. Small particles (such as less than 80 μm) can form a clay mixture by electrostatic action and intermolecular force in the liquid-phase excipient, but the morphology is unstable. Too small mineral powder can easily collapse and be absorbed in the tissue defect area. However, the small particle mineral matrix is conducive to the release of organic components^67–69^. Chitosan, silk fibroin and sodium alginate have been used as composite materials for bone tissue repair due to their osteoconduction property^70–72^. However, these composites are mostly preformed, lack injectability, and cannot be self-repaired after structural damage.

To improve the formability of TDM powder and expand its application range, we designed a TDM carrier with PAA as the NCP simulator and CMC as the glycosaminoglycan simulant (Figs. 1 and 2a–c). Sun and Lei first constructed the PAA/ACC mineral matrix gel with sodium carbonate and found that, for the formation of a stable gel state, the molecular weight of PAA must be at least 20,000^28,30,36^. However, TDM contains considerable protein, which mixes with the CO_2_ generated during the preparation, resulting in the formation of considerable foam. Meanwhile, the high PH value of sodium carbonate may affect the active substances in TDM. Thus, we selected the phosphate solution and promoted gel polymerization through *Ca*^*2+*^ addition; as a result, ACP was formed in the hydrogel^36,73^, as shown in Supplementary Fig. 1. ACP is involved in the mineralization of collagen fibrils. The existence of ACP can improve the structural stability of hydrogels^36,74^. Meanwhile, the dynamic networks of *Ca*^*2+*^*·COO*^*−*^ coordination bonds and ionic bonds were involved in the hydrogel’s noncovalent bond crosslinking and its self-healing and shear-thinning properties^36,75^. This can be proved by the FTIR spectrum (Fig. 4c). The change in the peaks of *υC*=*O* in PAA-ACP and PAA-CMC, contrasting with the PAA atlas, indicates that carboxylate is chelated with *Ca*^*2+*^ in both hydrogels^30^. The value of *δNH* in PAA-CMC shifted the offset compared to CMC, suggesting a charge effect between the amino in CMC and carboxyl in PAA^42^.

In clinical practice, appropriate maneuverability and low swelling ratio are more conducive to tissue repair and regeneration^76^. By strengthening hydrogen bonds or constructing a double-mesh hydrogel with multi-sacrificial bonds, the operability of the material can be maintained and the mechanical properties of the material can be improved^77,78^. Hydrogels based on hydrogen and ionic bonds can be easily affected by pH, temperature, and humidity, as well as the molecular weight and crosslinking degree of polymer materials. It is possible to adjust the hydrogel composition such that its swelling rate, water content, and degradation time, as well as its plasticity and hardness, are affected^79,80^. As CMC and PAA are mixed, they intertwine to form polymers based on electric charges, which increases the material’s hardness, promotes swelling reduction, and prolongs degradation (Fig. 3). The rheological characterization of mineralized hydrogel (Supplementary Fig. 5) also suggests that with the increase in CMC molecular weight, the value of *G*’ showed a certain upward trend, indicating that the material hardness increased. Tang et al.^80^ found that the improvement in the crosslinking degree and molecular weight of acrylic acid and CMC can affect the mechanical properties of the material. These results are in line with those obtained by related studies of hydrogels based on CMC and PAA^81,82^. As an amphoteric polymer material, the content and molecular weight of CMC can affect the ionic bond and hydrogen bond interaction with PAA, which may be one of the main factors affecting the swelling and rheological properties of the material^83^. Mineral substrates, such as TDM and nHA, can also increase the volume and hardness of hydrogels while improving their stability.

The swelling of materials in a liquid environment can affect their mechanical properties^84^. The rheological frequency sweeps of the hydrogels (Fig. 5) showed that, in the liquid environment, the linear viscoelastic interval of the hydrogels was shortened, the elasticity was gradually reduced, and the gels were swollen and liquefied. The three types of hydrogels continued to show the rheological characteristics of gel materials after swelling for 48 h; that is, within the viscoelastic range, they showed viscosity at low vibration frequency and elasticity at high vibration frequency^30,48^. After prolonging the soaking time of PBS-collagenase, the hydrogels degraded gradually. The degradation mode of the PAA-CMC material swelled and dissolved in the early stage, and the HA component could chelate with the carboxyl group to stabilize the gel structure. However, in the later stage, with the over-hydration of the gel, PAA-CMC-TDM eventually disintegrated and TDM particles were released into the environment^30^.

After an injury, stem cells play a critical role in tissue repair and self-renewal, and they function through paracrine, migration and differentiation^85,86^. DPCs are derived from nerve spines and are mainly found in the dental pulp tissue, which are essential for dental pulp tissue repair and regeneration^87,88^. In addition to odontoblast differentiation, DPCs are also capable of dividing into chondrocytes and osteoblasts. Scaffold materials combined with DPCs can promote bone repair in critical areas such as the mandible and skull^89,90^. Thus, DPCs can be used to evaluate the osteogenic effect of PAA-CMC-TDM. TDM demonstrates good biological activity and biocompatibility and can promote the differentiation of mesenchymal stem cells toward odontogenesis/osteoblast^16,91^. The use of CMC materials in tissue engineering spans a wide range, including bone defects and wound accessories^38^. Therefore, PAA-CMC-TDM materials can be used as bioactive materials for osteogenic induction.

The PAA-CMC-nHA group considerably affected the cell proliferation and migration to a considerable extent, although there was no statistical difference between the groups, according to the results of the CCK-8 and transwell/scratch experiments. From the perspective of composition, the extract of PAA-CMC-nHA contains PAA, CMC, nHA and rich calcium and phosphorus ions. The extract of PAA-CMC-TDM contained a large number of cytokines and NCPs from the natural extracellular matrix^16,92^. In PAA-CMC-nHA, PAA combines with the calcium of apatite to improve the stability of PAA-CMC, but there is no buffer medium for the release of free calcium and phosphorus ions. With the swelling and degradation of the materials, the concentration of *Ca* and *P* in the liquid environment increased. In some studies, extracellular *Ca*^*2+*^ have been found to promote the odontogenic and osteogenic differentiation of DPCs^93,94^. In addition, low concentrations of extracellular *Ca*^*2+*^ promote cell proliferation. In contrast, other studies have indicated that additional *Ca*^*2+*^ induce apoptosis of hDPCs^16,92,95,96^. However, the drastic change of ion concentration in extracellular environment may affect the normal physiological activities of cells and eventually lead to a decrease in cell activity^97^. In contrast, in PAA-CMC-TDM, DMP-1, OPN and other NCPs in TDM materials can chelate calcium ions, stabilize ACP, and enter the partially decalcified collagen structure to promote collagen remineralization, or directly participate in the mineralization of the extracellular matrix of cell differentiation, thus stabilizing the ion concentration in the environment^31^. Meanwhile, the nHA can be directly absorbed by cells after partial dissolution and can significantly promote osteogenic differentiation of cells^98^. However, it may also cause drastic changes in the ion concentration in the surrounding liquid environment, which may influence the vitality of cells^99^. At the same time, the apoptosis and increase in ROS induced by nanoparticles may affect cell activity^100,101^.

Alizarin red staining of PAA-CMC-NHA, PAA-CMC-TDM, and TDM showed obvious color change, suggesting that these materials may have suitable mineralization-inducing ability (Fig. 6a). In addition to the calcium nodules observed in TDM and PAA-CMC-TDM, the obvious staining might have been caused by mineral particles entering the cell-membrane sheets^96^. On this basis, we further discussed the change in the related indexes of hard tissue differentiation at the protein and RNA levels after 7 and 14 days of cell induction (Fig. 6b–f). There is evidence that TDM and its extracts affect dental pulp cell differentiation through Wnt/β-catenin signaling^102^. The RNA expression levels of OPN, DMP-1, and DSPP in hDPCs were significantly increased after 14 days of induction (PAA-CMC-nHA, PAA-CMC-TDM, and TDM) when compared to the control group. DSPP, DMP-1, OPN, and bone sialoprotein are members of the SIBLING family, which participate in the formation, maturation, and mineralization of dentinogenesis^103,104^. DMP-1 is an important protein involved in biomineralization. The N and C terminals of its protein structure impart polyelectrolyte properties, which allows it to stabilize ACP by binding calcium ions and guiding collagen fibers^105^. DSPP can be further transformed into DSP and DPP proteins, which regulates the formation of dentinal tubules^106^. OPN is a phosphorylated protein that has a high affinity for calcium and participates in the biological regulation of bone and dental mineralization. The polyaspartic acid residues and phosphorylated groups in OPN proteins may also contribute to the mineralization of collagen structures by forming and stabilizing ACP^107,108^. The upregulation of RUNX-2 expression was evident in the PAA-CMC-TDM group under 7-day induction. In osteogenesis, RUNX-2 is one of the most important transcription factors that activate osteoblast differentiation marker genes. As osteoblasts are differentiated, the expression of RUNX-2 increases in preosteoblasts, peaks in immature osteoblasts, and decreases in mature osteoblasts^109^. By binding to cis-acting elements in the promoter region, RUNX-2 can regulate some osteoblast-specific phenotypic marker expression levels (such as OPN and OCN)^110,111^. In addition, the ALP expression decreases in the groups that receive mineral materials inducers (PAA-CMC-nHA, PAA-CMC-TDM, and TDM). The expression activity of ALP is an obvious characteristic of osteoblast differentiation. Through the hydrolysis of inorganic pyrophosphate, ALP releases inorganic phosphate (Pi), providing the necessary phosphoric acid to form HA. However, high extracellular Pi content can inhibit ALP activity through a negative feedback mechanism. This is consistent with our results, considering the high phosphate content in the hydrogels prepared from mineral materials^112,113^. Chitosan-based materials can promote the regeneration of fibrous connective tissue and, thus, are often used in dressings to promote wound healing^114^. In our study as well, CMC exhibited a promoting effect on COL-I.

After the implantation of exogenous bone repair materials, neutrophils, macrophages, and other immune cells first enter the bone defect area. While promoting the degradation of the scaffold, they coordinate the recruitment of regeneration-related cells, and cooperate with the materials to promote new bone formation^115,116^. Assuming the PAA-CMC-TDM and TDM materials are implanted in defects, similar phenomena are likely to occur. For an intuitive evaluation of PAA-CMC- TDM’s inflammatory response, we implanted it directly into the subcutaneous tissue. The TDM particles were wrapped by hyperplastic fibrous connective tissue 14 days after the material was injected, as shown in Fig. 7a, b. The immunofluorescence results showed that CD68+ (M1 macrophage marker) and CD163+ (M2 macrophage marker) macrophages were infiltrated at the interface of the materials and surrounding tissues on the 7th day, but the macrophage reaction was not evident after 14 days, and the expression levels of CD68 and CD163 were decreased^117^. Under appropriate stimulation, macrophages were activated into a state of inflammation and can be roughly divided into two types: M1 (classical activation) and M2 (alternative activation). M1-type macrophages exhibit pro-inflammatory effects associated with immune responses to both bacterial and intracellular pathogens. In contrast, M2-type macrophages exhibit more anti-inflammatory effects and have related functions in angiogenesis and wound healing^118,119^. By polarizing macrophage M1/M2 to reduce absorption, biological tooth root composites developed from TDM can improve the retention rate and stability after implantation^120,121^. These results indicate that PAA-CMC-TDM, as a substitute for tissue repair, showed low immunogenicity and suitable biocompatibility.

Dentin and bone tissue have similar chemical compositions (collagen fibers and HA) and are developed from the mesenchymal cells of mesoderm. As bones develop, they are formed by two osteogenic mechanisms: intramembranous ossification and endochondral ossification. Dentin is developed by spherical mineralization after the formation of collagen fibers^56,122,123^. According to the morphological structure, bone can also be classified into long bone, flat bone and irregular bone^22^. The femur is a long bone and has a classical bone structure, including trabecula, bone marrow cavity, and cortical bone. Its blood supply mainly originates from the peripheral blood vessels and the internal bone marrow cavity. The skull is a flat bone, which is similar to other maxillofacial bone structures, with vascular structure inside and rich trabeculae on the surface, but no definite bone marrow cavity structure^124,125^. Unlike bone, teeth are directly exposed to the oral environment, and their internal vascular network only interacts with the outside through the root canal orifice; thus, their ability to self-repair is limited^4,126^. The existence of vital pulp forms the basis for maintaining the biological activity of the pulp–dentin complex and is vital for the preservation and continuous development of young permanent teeth whose apical foramen is not completely closed^127^. Leveraging the origin and ability of TDM to repair hard tissue, we aim to develop multi-purpose repair materials. To evaluate the ability of PAA-CMC-TDM and TDM materials to repair hard tissue defects, a skull defect model, femur defect model and dental pulp defect model were selected.

Zhao et al.^30^ constructed mineralized CHAp-PAA hydrogels with sodium carbonate, nHA and PAA, and proved that these hydrogels have good bone repair ability in rabbit femoral defects. However, in this research, after CMC addition, the PAA-CMC-nHA group did not achieve better results than TDM in vitro. Based on previous cytological experiments, we compared the repair of hard tissue defects among PAA-CMC-TDM, TDM, and blank treatments in animal experiments. In the femoral defect (Fig. 8), the defect area of the TDM group was healed after 6 weeks, while some particles remained in the PAA-CMC-TDM group. PAA-CMC-TDM showed almost complete healing of the defect area in skull defects, while TDM showed evident new bone tissue formation, partially healing the defect (Fig. 9). The defect of the constructed femur was not completely transfixable, which was beneficial to the implant’s preservation. In contrast, the functional components per unit volume in the defect area of the TDM group were more than those in the PAA-CMC-TDM group, and the repair effect was more evident^22^. The calvarial defect we prepared was 5 mm in diameter and exposed the penetrating defect of the dura mater, which is a critical bone defect. In this case, PAA-CMC-TDM exhibited good stability and filling ability, which can ensure the stable retention and function of TDM functional components to achieve a better effect of bone repair^128^.

The preservation treatment of the dental pulp partially damaged by accident or infection depends on the interaction between the pulp capping material and dental pulp tissue, forming a reparative dentin bridge. This process involves the early inflammatory reaction after pulp capping, the induction of homing of mesenchymal stem cells, and the differentiation of dentin^129^. As a type of widely used commercial pulp capping material, i Root BP plus is an improved calcium silicate (CSC)-based material, with the main components of calcium silicate, zirconia, tantalum oxide, and calcium sulfate^130^. As a strong alkaline vital pulp preservation material, i Root BP plus solidifies in water and releases *OH*^*−*^, *Ca*^*2+*^, *Si*^*4+*^, and other components to achieve antibacterial, stem-cell differentiation and dentin bridge formation^131,132^. Compared to CH, CSC exhibits better bioactivity, forms intact and dense dentin bridges, and achieves excellent efficacy in long-term clinical observations^133–135^. In this study, i Root BP plus was introduced as a positive control to evaluate the ability of PAA-CCMC TDM to promote restorative dentin formation through the Beagle dog pulp defect model. Based on the histological and imaging methods, TDM and its derivatives promoted the formation of restorative dentin. Moreover, the scaffold (PAA-CMC-TDM) prevented the root canal calcification caused by TDM particles. Holiel et al.^17^ achieved the vital pulp therapy of human third molars after pulp penetration by using TDM derived from autologous teeth. Furthermore, they compared the therapeutic effects of direct pulp capping of TDM, Biodentine and MTA in patients with accidental traumatic pulp exposures. According to the CBCT results, there were no significant differences among the three groups^136^. According to some histological studies, the mineralized dentin bridge of the CSC material is different from the dentine bridge. The biological process is more likely to be considered a reparative process than a regeneration response^137^. Meanwhile, the mild or moderate inflammation induced by CSC to stimulate the regenerative process is concerning. Strongly alkaline materials are beneficial to control infection, but can also lead to complications such as root canal hypercalcification and root resorption^138^.

In summary, based on biomimetic mineralization, we used PAA- and CMC-chelated *Ca*^*2+*^ to assemble supramolecules independently to construct a bioactive mineral gel PAA-CMC-TDM with the dentin matrix as the mineralization core template. Based on noncovalent bond networks, such as ionic, hydrogen, and metal coordination bonds, PAA-CMC-TDM was found to be moldable, injectable, and self-healing, and exhibited suitable initial stability in the physiological environment. The synthesized PAA-CMC-TDM could maintain the bioactivity of TDM and induce osteogenic and odontogenic differentiation of DPCs. Furthermore, the results of animal experiments showed that our developed PAA-CMC-TDM can promote in situ dentin/bone regeneration in complex defect areas, which can be used as a potential scheme for clinical bone repair.

## Methods

### Materials

HA (~97%, <100 nm particle size), sodium carbonate (Na_2_CO_3_, ~99.5%), sodium phosphate dibasic dihydrate (Na_2_HPO_4_·2H_2_O, ~98%), calcium chloride (CaCl_2,_ ~96%), calcium hydroxide (Ca(OH)_2_, ~95%), and sodium hydroxide solution (NaOH, 1.000 mol/L) were purchased from Aladdin. Carboxymethyl chitosan (CMC, Mw ≈ 20,000, 60,000, 100,000, and 150,000) was purchased from BoMei Biotechnology (Hefei, China). Ethanol, potassium chloride (KCl), hydrochloric acid (HCl, 36.0–38.0%), and sodium chloride (NaCl) were purchased from Kelong (Chengdu, China). PAA (Mw ≈ 250,000, 35 wt%), 6-diamidino-2-phenylindole (DAPI), β-sodium phosphate glycerol, 1,25-dihydroxyvitamin D3, alizarin red, vitamin C, ethylene diamine tetra-acetic acid (EDTA) and dimethyl sulfoxide were obtained from Sigma-Aldrich. Sodium pentobarbital, sterile syringe filter (33 mm diameter, 0.22 µm pore size hydrophilic polyether sulfone membrane) were obtained from Merck Millipore (Germany). Paraformaldehyde, Tween-20, Triton X-100, PBS, Collagenase-I and Hematoxylin-Eosin were obtained from Biosharp (Guangzhou, China). In addition, i Root BP Plus was obtained from Innovative Bioceramix (Canada). The α-minimal essential medium (α-MEM), fetal bovine serum (FBS), and penicillin/streptomycin solution were purchased from Hyclone (USA). Transwell chambers with 8-μm-pore polycarbonate membranes were purchased from Corning (New York, NY, USA). CCK-8, BCA Protein Assay Kit, and Total Protein Extraction Kit were obtained from KeyGen Biotech (Nanjing, China). Cell/Tissue Total RNA Isolation Kit (Lot#RC112-01), cDNA Synthesis Kit (Lot#R312-01), and SYBR qPCR Master Mix (Lot#Q712-02) were obtained from Vazyme (Nanjing, China). Masson’s Trichrome Stain Kit and the Hematoxylin-Eosin/HE Staining Kit were obtained from Solarbio (Beijing, China). The PAGE Gel Fast Preparation Kit (10%, Lot#PG112) was obtained from Epizyme (Shanghai, China). The antibodies used in this experiment are listed in Supplementary Table 2. The Clarity Western ECL Substrate Kit was purchased from Bio-Red (USA).

### Ethics approval statement

The experimental protocol for the animal experiments and the acquisition of related human biological samples have been approved by the Research Ethics Committee of West China Hospital of Stomatology (The West China Hospital of Stomatology Institutional Review Board, WCHSIRB-D-2021-414, WCHSIRB-CT-2021-362).

The hDPCs and the dentin matrix were derived from healthy third molars extracted for clinical reasons at West China Hospital of Stomatology. Human samples in this study were obtained with informed consent from all donors and under the supervision and guidelines of the West China Hospital of Stomatology Institutional Review Board (WCHSIRB-CT-2021-362).

The female SD rats and female beagle dogs were obtained from Dashuo Experimental Animal Center (China). Animals were housed in ventilated cages on a 12:12-h light/dark cycle with ad libitum access to food and water.

### Preparation of TDM materials and mineralized hydrogels

We prepared the TDM material through human-and pig-derived isolated teeth based on previously proposed methods^16,18^. After the periodontium, the dental crown, dental pulp, and predentin were removed; the teeth’s residual tissue was demineralized using EDTA gradients (17%, 10%, and 5%), and cleaned and freeze-dried to obtain TDM. TDM was repeatedly milled and screened with a frozen grinding machine until the diameter of the obtained powder did not exceed 40 μm.

The hydrogel was prepared at room temperature. First, aqueous solutions of CaCl_2_ (0.1 mol/L, 5 mL) and PAA (35 wt%, 1 mL) were mixed to form a transparent precursor solution, named “solution A”. Next, according to the experimental design, a certain amount of CMC was dissolved in the aqueous solution of Na_2_HPO_4_ (0.2 mol/L, 10 mL) to obtain a uniform white suspension supplemented with TDM or nHA, named “solution B”. Under vigorous stirring, “solution B” was added dropwise to “solution A” until a uniform white suspension was formed. After stirring the suspension at 600 rpm for 24 h, CaCl_2_ (1 mol/L) was added to the solution, and the hydrogel gradually clustered around the Teflon-coated stirrer. The hydrogel was then washed three times with deionized water and sterilized with ethylene oxide after 8–12 h of lyophilization. After the same amount of hydrogel was freeze-dried, the sample’s contact angle was measured using a MobileDrop contact angle meter (Kruss, Hamburg, Germany) to determine the surface hydrophilicity.

To observe the material’s stability in liquid, we took the TDM powder lump as the control group and PAA-CMC-TDM as the experimental group, both of which were placed in PBS and ultrasonically shaken for 5, 10, and 15 min to observe the material’s shape. Then, PAA-CMC-TDM was dyed blue and red with pigments, respectively, and reassembled and left at room temperature for 30 min to observe the self-healing of the hydrogel. The 0, 10 and 30 min of recombinant hydrogels were respectively taken, lyophilized and then SEM was used to observe the healing of the section.

### Swelling properties and relative water content

To test the relative water content and swelling ratio, the hydrogels were equilibrated in PBS for 24 h at 37 °C. After cleaning, the hydrogel was freeze-dried for 12 h to determine the water content. The determination of the swelling degree of hydrogels required the material to be incubated in PBS for 48 h.

The equilibrium water content was calculated as follows:

Equilibrium water content (%) = (*W*_*w*_ – *W*_*d*_) / *W*_*w*_ × 100% (*W*_*w*_ and *W*_*d*_ indicate the weights of wet and dry hydrogels, respectively)

The swelling ratio of the hydrogels was quantified as follows:

Swelling ratio (%) = (*W*_*s* 48h_ / *W*_*d*_) × 100% (*W*_*s* 48h_ indicates the swollen weight for 48 h in PBS and indicates dry weight)

To obtain more accurate results, these experiments were performed in triplicate.

### Degradation of PAA-CMC hydrogels

The degradation of the PAA-CMC hydrogels was conducted for 12 days. These hydrogels were incubated at 37 °C in PBS with 2 U/mL collagenase and replaced every 3 days. The hydrogels were lyophilized, and the weight was determined as follows:

Residual weight (%) = (*W*_*t*_ / *W*_0_) × 100% (*W*_0_ and *W*_*t*_ indicate the hydrogel weights at times 0 and *t*, respectively)

### Component and structure characterization of hydrogels

SEM (Inspect F, FEI, USA) was applied to observe the surface and internal structure of the hydrogels. Briefly, hydrogels of different compositions were lyophilized and cut off after embrittlement in liquid nitrogen, sprayed with gold on the surface and cross-section, and then placed under a scanning electron microscope for observation. The elemental *Ca* and *P* analyses and sample mapping were performed using an EDAX super octane (Apreo 2C, Thermo Fisher Scientific, USA).

The morphology of calcium phosphate nanoparticles in PAA-CMC hydrogels without TDM and nHA was observed using a transmission electron microscope (Hitachi H7500, Japan). The hydrogel was freeze-dried, ground into 40 μm powder, and resuspended with deionized water at a 1 mg/mL ratio. The mixture was coated on the copper mesh surface and left to dry in a natural state, and the micromorphology of the sample was observed at a voltage of 200 kV.

The chemical composition and status of PAA-CMC hydrogels were determined using an XPS (Thermo Fisher ESCALAB Xi+ XPS System). XPS data were processed using the AVANTGE software.

FTIR spectroscopy was used to characterize the materials. The raw material PAA, CMC, TDM, and nHA and the prepared PAA-ACP, PAA-CMC-ACP, PAA-CMC-nHA, and PAA-CMC-TDM hydrogels were subjected to vacuum-drying to obtain a solid powder and characterized on a Thermo Fisher Nicolet Is5 (FTIR) within a scanning range of 4000–400 cm^−1^.

The crystal types of dry mineral hydrogels were analyzed using a Bruker D8 Advance XRD (Bruker, Darmstadt, Germany). Regarding the test sample powder on the glass substrate, *Cu-Kα* was used as the radiation source at 40 kV voltage with an operating current of 40 mA, scanning rate of 0.02°, and scanning range of 10–90°.

The TG-DTG of PAA-ACP, PAA-CMC-ACP, PAA-CMC-nHA, and PAA-CMC-TDM was conducted using a Mettler Toledo TGA2 thermobalance (Columbus, USA). The test temperature ranged from 50 to 800 °C, the heating rate was 10 °C/min, and the purging gas was air.

### Rheological characterization

A rheometer (DHR-1, TA Instruments, USA) was used to evaluate the rheological properties of PAA-CMC, PAA-CMC-nHA, and PAA-CMC-TDM hydrogels with different compositions and concentrations. All tests were conducted in the oscillatory mode, at 25 °C, using a flat rotor of 25 mm diameter. First, we evaluated the rheological properties of the hydrogels after they were subjected to partial composition changes using vibration time scanning at a fixed stress of 10.00 Pa and frequency of 50.00 Hz. Then, under controlled other variables, we adjusted the content of TDM or nHA and molecular weight and content of CMC, and measured the *G’* and *G”* values of the different hydrogels. In addition, the rheometer was used to test the following three rheological properties of the hydrogels: (1) the hydrogels’ linear viscoelastic region, storage modulus, and loss modulus (10 Hz, 0.1–100% strain), which were determined using the strain sweep method; (2) the hydrogel modulus under the frequency scanning tests (10 Pa stress, 0.1–50 Hz); and (3) the strain-induced damage and healing of the hydrogels (10 Hz, strain of 100% and 0.1% for 200 s).

### Cell culture of human dental pulp stem cells

The hDPCs were derived from healthy third molars. The pulp tissue was minced with sterile scissors and subjected to enzymatic digestion with 1 mg/mL type-I collagenase for 30 min and tiled on the bottom of a culture bottle^52^. The DPCs were cultured in α-MEM supplemented with 10% FBS and 1% (v/v) penicillin/streptomycin solution at 37 °C in 5% CO_2_. The vimentin expression was analyzed by immunofluorescence staining following the manufacturer’s protocol. Immunostaining was performed using primary antibodies against vimentin and the secondary antibody Alexa Fluor 488. After counterstaining the nuclei with the DAPI reagent, the cells were examined under a fluorescence microscope (Leica Optical, Germany). The hDPCs in passages 2–5 were used for the experiments.

### Chemotaxis assay of hDPCs with mineralized hydrogel

We used transwell assay and scratch wound‐healing migration assay to evaluate the cell chemotaxis. For the transwell assay, hDPCs (1 × 10^4^) were seeded into the upper compartment of the chambers. The extracts of TDM, PAA-CMC-nHA, PAA-CMC-TDM, and CMC were coated on the bottom in the lower compartment. After 24 h, cells migrating to the lower side of the filter were fixed with 4% paraformaldehyde for 30 min, stained with crystal violet for 20 min, and counted under a microscope in six predetermined fields using Image J software.

For the scratch wound assay, the cells were seeded in dishes and a scratch was created in the cell monolayer with a 1000 µL pipette tip. The scratch area was photographed immediately after the scratch was created and after 48 h, and was measured using Image J software. Cell migration rate (%) = (1 – scratch area/original scratch area) × 100. All experiments were independently repeated three times.

### Cell proliferation ability of hDPCs with mineralized hydrogels

The effects of the hydrogel on hDPC proliferation were evaluated using the CCK-8 (Dojindo, Japan). The hDPCs with a density of 5 × 10^3^ cells per well were cultured in 96-well plates and the cells were kept in TDM, PAA-CMC-nHA, PAA-CMC-TDM, and CMC extracts. All steps were performed according to the manufacturer’s protocol. The OD value was measured using the Multiskan Go spectrophotometer (Thermo Fisher Scientific, USA).

### Alizarin red S staining

The osteogenic medium comprised α-MEM, 10% FBS, 5 mM L-glycerophosphate (Sigma, USA), 100 nM dexamethasone (Sigma, USA), and 50 mg/mL ascorbic acid (Sigma, USA). To prepare the conditioned medium, the materials (TDM, PAA-CMC-nHA, and PAA-CMC-TDM) were incubated with α-MEM with a weight-to-volume ratio of 1 g/10 mL, and the conditioned medium of CMC was 10 mg/mL. The hDPCs with logarithmic growth of P3 were seeded into 6-well plates at 1 × 10^5^/well. Under 80% confluence, the culture medium was replaced with the osteogenic medium. When induction was complete, the osteogenic medium cells were stained with 0.1% ARS solution (Sigma) and photographed under a phase-contrast inverted microscope (Nikon, Japan).

### Real-time quantitative polymerase chain reaction

The hDPCs seeded at 10^5^ cells per well in 6-well plates were cultured in the extract of TDM, PAA-CMC-nHA, PAA-CMC-TDM, and CMC for 7 and 14 days. The total ribonucleic acid of the induced cells was extracted with the Cell/tissue Total RNA Isolation Kit and reverse-transcribed into cDNAs through the cDNA Synthesis Kit. Real-time quantitative polymerase chain reaction (RT-qPCR) was performed using the LightCycler PCR System (Roche) with SYBR qPCR Master Mix. The cycle threshold (Ct) was recorded after each reaction and used in the 2^–ΔΔ^ Ct method to calculate the relevant date with GAPDH as the loading control gene. The primer sequences are listed in Supplementary Table 1. All experiments were performed in triplicates.

### Western blot analysis

The experimental procedure followed the manufacturer’s instructions provided with the PAGE Gel Fast Preparation Kit (10%). Briefly, the hDPCs seeded at 10^5^ cells/well into 6-well plates were cultured in the extracts of TDM, PAA-CMC-nHA, PAA-CMC-TDM, and CMC for 7 and 14 days. The total protein of cells in all groups was extracted using the Total Protein Extraction Kit and resolved on 10% polyacrylamide gels, which were blotted onto a nitrocellulose membrane. After blocking with 5% skimmed milk, the membrane was incubated first with the primary antibody and then with the secondary antibody. The chemiluminescent protein was visualized with an ECL reagent (GE, USA). The antibodies used to detect the target protein mainly included COL-1, ALP, DMP-1, RUNX-2, DSPP, OPN, GAPDH (Supplementary Table 2). The thoroughly rinsed membrane was incubated with the secondary antibody coupled to the species-matched HRP. The band intensity was quantified using NIH Image J software. The experiment was repeated three times independently. The uncropped scans of the blots shown in the manuscript were supplied in Supplementary Figs. 9 and 10.

### In vivo biocompatibility study of rats

Female SD rats (200–250 g) were used for the in vivo experiments. After injecting anesthesia with sodium pentobarbital, an incision was made along the median line of the back skin of the rats, and connective tissues were spread to create a subcutaneous pocket on both sides of the wound. Next, PAA-CMC-TDM hydrogels were implanted into the subcutaneous pockets. After 7 and 14 days of implantation, the rats were euthanized by excessive pentobarbital injection. The materials’ skin tissues were collected for H&E staining and fluorescent immunohistological staining of CD68 and CD163. Each group comprised three rats.

### In vivo study of small animal model bone regeneration

Female SD rats (250–350 g) were used in this study. To explore the ability of the mineralized hydrogels for bone defect repair, we used a rat calvarial and a femoral bone defect model to evaluate the osteoinduction of the composite hydrogels in vivo. All animals were randomly assigned to three and six weeks with bone defect models.

After injecting anesthesia with sodium pentobarbital, 16 rats received a drill hole defect (Ø 2.5 mm, depth 3 mm) with a high-speed dental handpiece and a small ball drill (Ø 1 mm) under constant cooling with NaCl in the lateral femoral condyle of the left hindlimb, and PAA-CMC-TDM (*N*_3 week_ = 4, *N*_6 week_ = 4) or TDM (*N*_3 week_ = 4, *N*_6 week_ = 4) was filled in the defect according to the experimental design. The same defect was prepared in the rat’s right femur as a control group. After the surgery, the skin was stitched to close the defect.

Similar to the above procedure, after injecting anesthesia, the surgical area of the rat head was shaved and disinfected, and the skin was cut longitudinally to expose the skull. With a drill bit, two craniotomy defects (diameter 5 mm) were formed on the parietal bone of the skull on both sides of the sagittal suture line. The defects were washed with saline, PAA-CMC-TDM (*N*_3 week_ = 4, *N*_6 week_ = 4), or TDM (*N*_3 week_ = 4, *N*_6 week_ = 4) filled in the left defect, and the right defect was used as the control group. Then, the soft tissue was closed with stitches.

### Beagle dog’s pulp repair model in vivo

Three female beagle dogs (15 kg) were used in this study. All experimental teeth of each dog were divided into the following three groups: PAA-CMC-TDM (left maxillary region, *N*_6 week_ = 8 teeth), i Root BP plus (right maxillary region, *N*_6 week_ = 8 teeth), and TDM (right mandibular region, *N*_6 week_ = 8 teeth). All experimental subjects were anesthetized with sodium pentobarbital, which was injected intramuscularly.

Using sterile saline as a coolant, a 2-mm-diameter hole was perforated on the tongue/occlusal surface in the experimental teeth with a high-speed dental handpiece. After using direct pulp capping material as the experimental material, the perforation was sealed with glass ionomer cement (Fuji, Japan). The samples were collected after 6 weeks and fixed with 4% neutral-buffered formalin for 1 week.

### Histological analysis and micro-computed tomography imaging evaluation

A micro-computed tomography scanner (Skyscan 1276, Kontich, Belgium) was used to quantify the bone parameters of the femurs (10 µm, 70 kV, 357 uA), skulls (10 μm, 70 kV, 200 uA), and teeth of beagle dogs (20 μm, 100 kV, 200 uA).

Then, the samples were demineralized in 10% EDTA solution for three months. After dehydration and being embedded in paraffin, the demineralized samples were cut into 5-mm-thick sections. Finally, they were subjected to H&E and Masson’s trichrome staining to determine the extent of mineralization tissue formation.

### Statistical analysis

The results of at least three independent experiments, performed using GraphPad Prism v8.0 and Origin 2019, were obtained and presented as mean ± standard deviation. The differences between two and multiple groups were compared by two-tailed Student’s *t*-test and one-way analysis of variance. A value of *p* < 0.05 was considered statistically significant.

### Reporting summary

Further information on research design is available in the Nature Research Reporting Summary linked to this article.

## Supplementary information

Supplementary Material

Reporting Summary

## Data Availability

The datasets generated during and/or analyzed during the current study are available from the corresponding author on reasonable request.

## Code availability

No custom code or mathematical algorithm was used in this study. Experimental data statistics in this study were performed using GraphPad Prism v8.0 and Origin 2019, and obtained and presented as mean ± standard deviation. The differences between two and multiple groups were compared by two-tailed Student’s *t*-test and one-way analysis of variance. A value of *p* < 0.05 was considered statistically significant.
