# Supplementary material for: Biomineralization-inspired mineralized hydrogel promotes the repair and regeneration of dentin/bone hard tissue
Source: NPJ Regen Med. 2023 Feb 25;8:11. doi: 10.1038/s41536-023-00286-3 (PMC9968336; doi:10.1038/s41536-023-00286-3)
Supplement: Supplementary file 1 — Supplementary Material [file 41536_2023_286_MOESM1_ESM.pdf]

Supplementary Materials

Biomineralization-inspired mineralized hydrogel promotes the repair and regeneration of dentin/bone hard tissue

Bo Wen[1, 2, 3, 4], Yuguo Dai[1, 2, 3], Xue Han[1, 2, 3], Fangjun Huo[1,2], Li Xie[1,2], Mei Yu[1,2], Yuru Wang[1, 2, 3], Ning An[1, 2, 3], Zhonghan Li[1, 6], Weihua Guo[1, 2, 3, 5*]

Corresponding author:

*Corresponding author, Department of Pediatric Dentistry, West China College of Stomatology, Sichuan University, No.14, 3rd Section, Renmin South Road, Chengdu 610041, PR China; Yunnan Key Laboratory of Stomatology, The Affiliated Hospital of Stomatology, School of Stomatology, Kunming Medical University, Kunming, Yunnan, PR China. Tel/fax: +86 28 8550 3499. Email address: guoweihua943019@163.com

Supplementary table 1 The primer sequences used for RT-qPCR

Gene	Forward sequence/reverse sequence
GAPDH	5`- TCAGCAATGCCTCCTGCAC-3`/
	5`- TCAGCAATGCCTCCTGCAC-3`
DMP-1	5`- AGAAGCGAGCTTGATGACAACAA-3`/
	5`- TGGACTCACTGCTGGGACCATCTAC-3`
ALP	5`- ACATTCCCACGTCTTCACATTT-3`/
	5`- AGACATTCTCTCGTTCACCGCC-3`
DSPP	5`- ATATTGAGGGCTGGAATGGGGA-3`/
	5`- TTTGTGGCTCCAGCATTGTA-3`
RUNX-2	5`- ATGAAATGCTGGAGTGATGTGG-3`/
	ATGAAGCCTGGCGATTTAGAGT-3`
OPN	5`- CAGTTGTCCCCACAGTAGACAC-3`/
	5`- GTGATGTCCTCGTCTGTAGCATC-3`
COL-I	5`- GAGGGCCAAGACGAAGACATC-3`/
	5`- CAGATCACGTCATCGCACAAC-3`

Supplementary table 2 The antibodies list

Antibody	Provide supplier	Catalogue numbers	dilutions
ALP	HUABIO (China)	ET1601-21	1:1000
RUNX-2	HUABIO (China)	ET1612-47	1:1000
OPN	HUABIO (China)	0806-6	1:1000
COL-I	HUABIO (China)	ET1609-68	1:1000
GAPDH	HUABIO (China)	ET1601-4	1:5000
DMP-1	Novus Biologicals (USA)	NBP1-45525	1:1000
CD68	Abcam (UK)	ab125212	1:200
Vimentin	Abcam (UK)	ab92547	1:200
CD163	Abcam (UK)	ab182422	1:200
DSPP	Zen Bioscience (China)	508413	1:1000
Goat anti-rabbit IgG-HRP	Zen Bioscience (China)	511203	1:5000
Goat anti-mouse IgG-HRP	Zen Bioscience (China)	511103	1:5000
Alexa FluoR 488 Goat anti-Mouse	Invitrogen (USA)	A31627	1:200
Alexa FluoR 555 Goat anti-Rabbit	Invitrogen (USA)	A31627	1:200

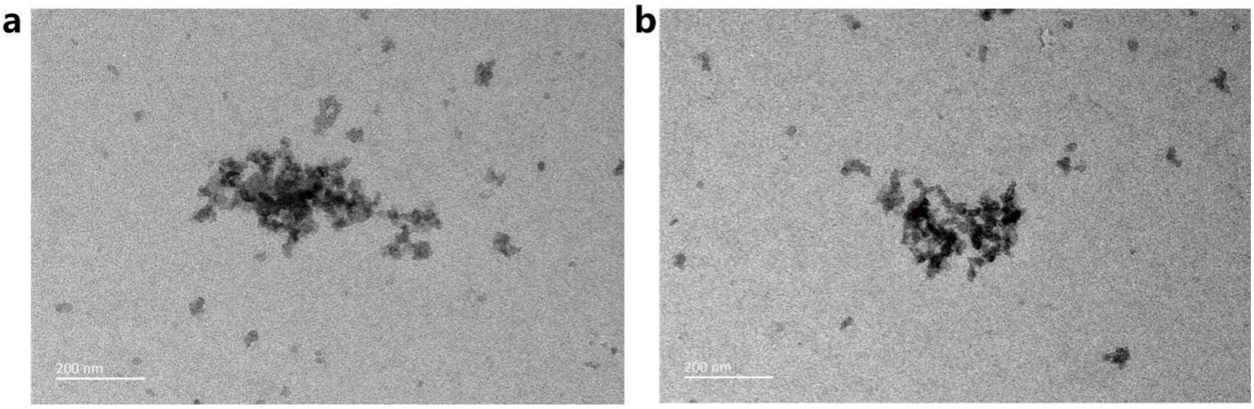

Supplementary Figure 1 TEM for PAA-CMC-ACP. Transmission electron microscopy (TEM) demonstrated black high-density particles located within PAA-CMC, which was similar to those reported in the literature about amorphous calcium phosphate (ACP).

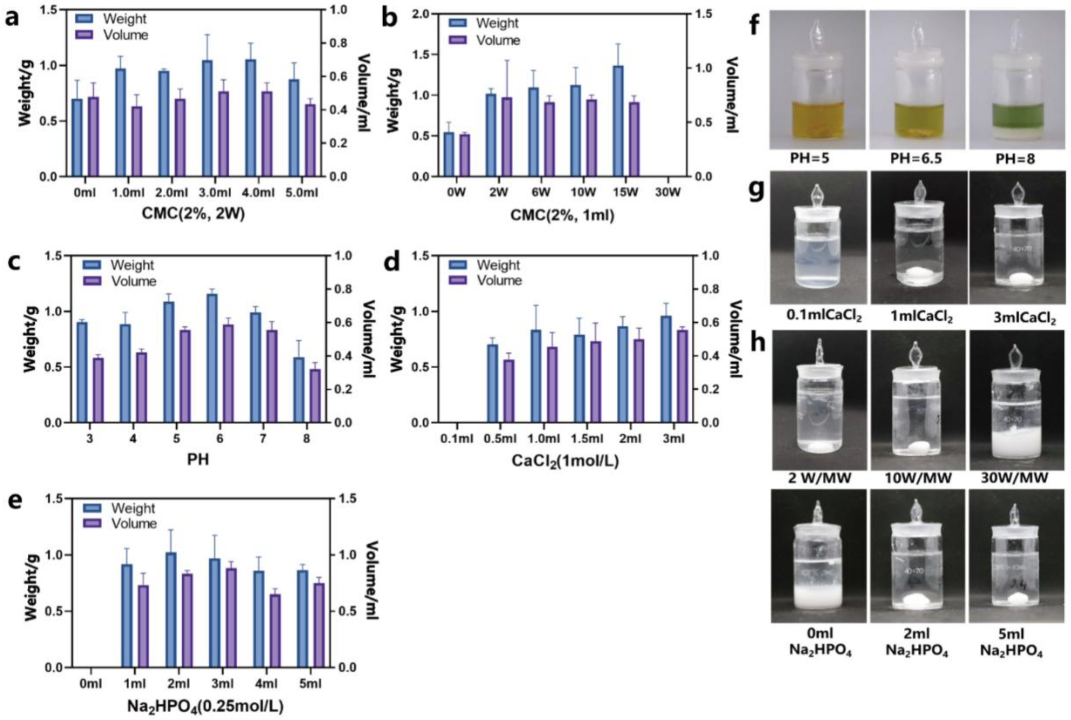

Supplementary Figure 2 Mass and volume changes after adjusting material composition and preparation conditions. It can be found that when other preparation conditions are consistent, the hydrogel material is difficult to form when PH ≥ 7.5. Similarly, when the molecular weight of CMC is 30 W, the formation is an unformed flocculate, just like the control group without Na_2HPO_4. At the same time, a certain amount of $CaCl_2$ (≥ 0.5 mL, 1mol/L) can make the gel polymerize and settle. **a–e** Optical pictures of material preparation system with different molecular weight of CMC **(a)**, different content of CMC **(b)**, different PH **(c)**, different content of $CaCl_2$ **(d)**, and different content of Na_2HPO_4 **(e)**. **f–h** Partial display of the preparation of hydrogels with different components.

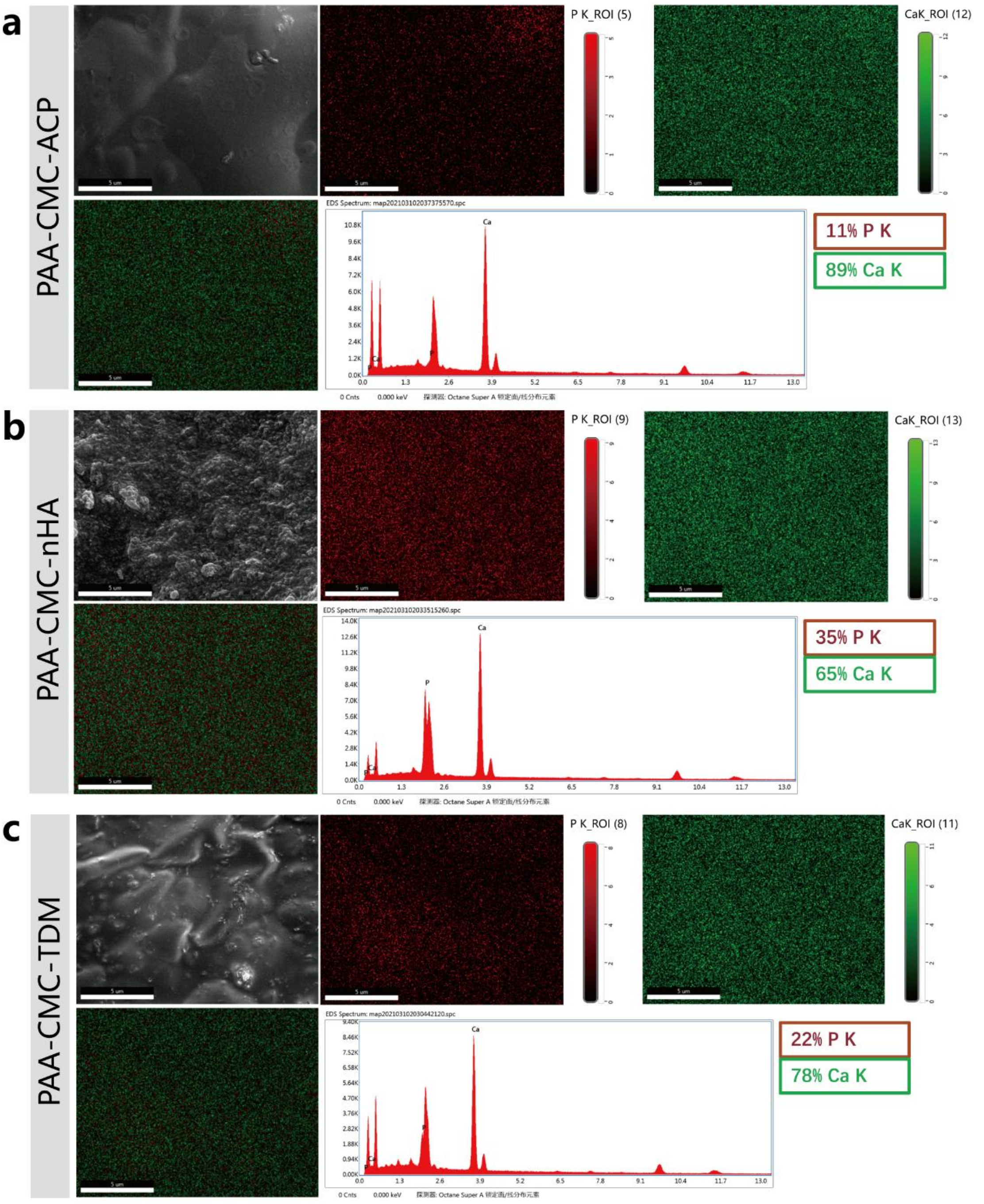

Supplementary Figure 3 SEM analysis coupled with EDX mapping (STEM-EDX) of the hydrogel. SEM analysis coupled with EDX mapping (STEM-EDX) displays the content and distribution of calcium and phosphorus ions on the hydrogel surface. **a** PAA-CMC. **b** PAA-CMC-nHA. **c** PAA-CMC-TDM.

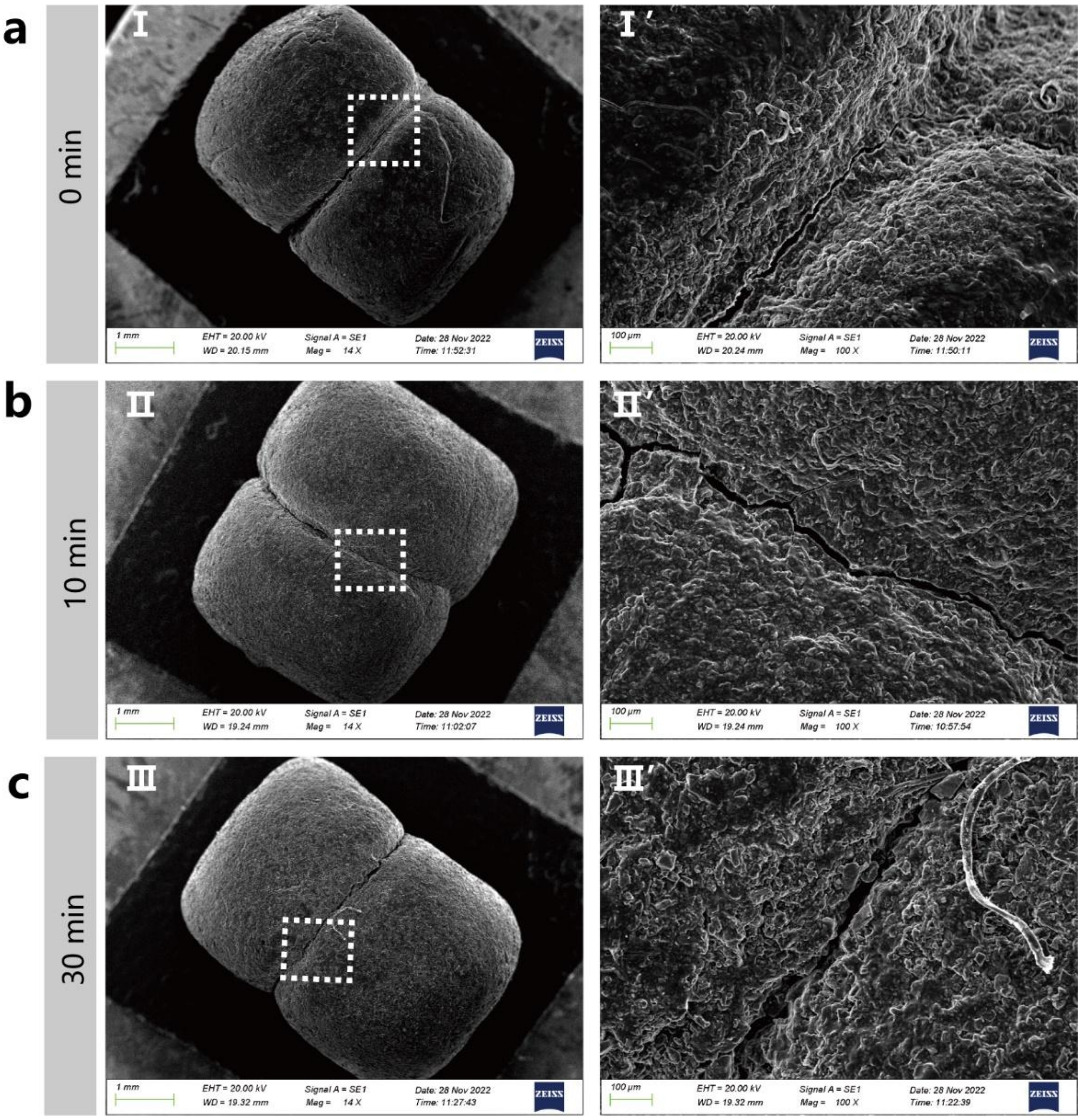

Supplementary Figure 4 SEM analysis of hydrogel self-healing experiment. The separated hydrogels were reassembled and freeze-dried in the initial stage **(a)**, recombinant 10 min **(b)** and recombinant 30 min **(c)**, respectively, and the interface of the recombination was observed by SEM**.** Scale bar, (I, II, III): 1 mm; (I', II', III'): 100 μm.

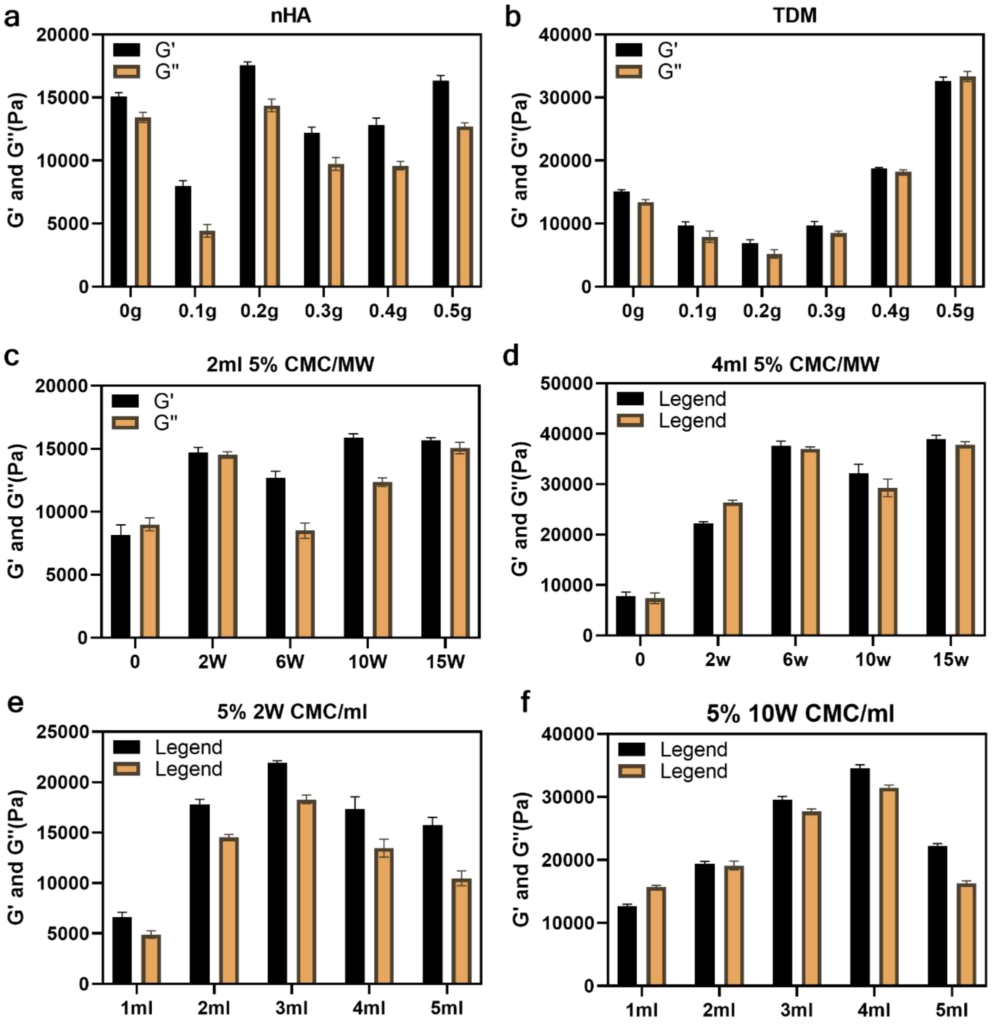

Supplementary Figure 5 Changes of material storage modulus (G') and loss modulus(G") after adjusting material composition. **a–b** Under the condition of 5% CMC (2W MW, 2 mL), the amount of TDM/nHA is adjusted from 0.1 g-0.5 g. **c–d** Under the condition of 5% CMC (**c** 2 mL, **d** 4 mL) and 0.1 g TDM, adjusting CMC molecular weight (2W, 6W, 10W, 15W). **e–f** Under the condition of 5% CMC (**e** 2W MW, **f** 10W MW) and 0.1 g TDM, adjusting the content of CMC (1 mL–5 mL).

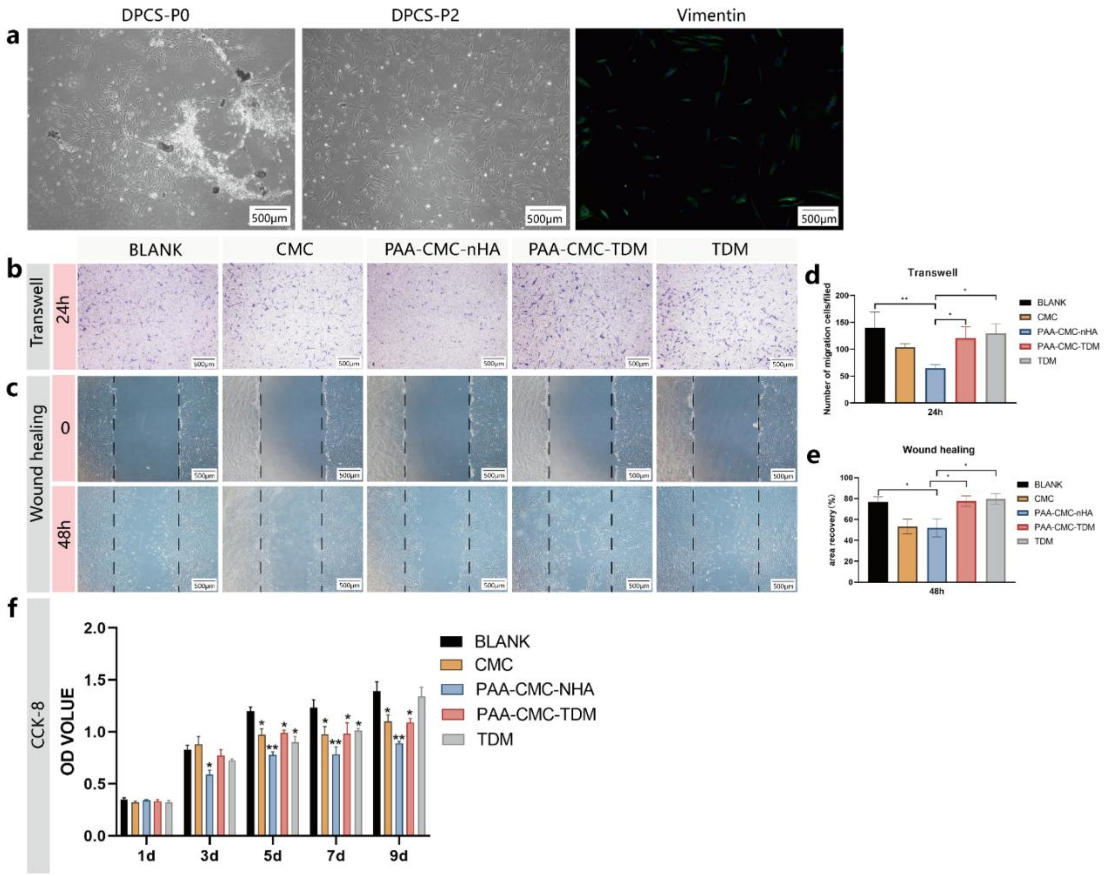

Supplementary Figure 6 Effect of hydrogel on the proliferation and migration capacity of DPCs. **a** The primary DPCs were obtained with the typical morphologic characteristics of mesenchymal cells in fibroblastic and spindle-shaped; hDPCs were positive for Vimentin. **b** Transwell migration assay with the extract of TDM, CMC and mineralized hydrogels. **c** Cell migration (scratch) assay for 48 h with the extract of TDM, CMC and mineralized hydrogel. **d** Quantification of transwell migration assay data. **e** Quantified cell migration (scratch) assay data. **f** Proliferation of DPCs after treatment with the extract of TDM, CMC and mineralized hydrogels, as measured by CCK-8. Error bars indicate standard deviation ($n = 3$); $*p < 0.05$, $**p < 0.01$, and $***p < 0.001$ (two-way ANOVA)**.** Scale bar, (a, b, c): 500 μm.

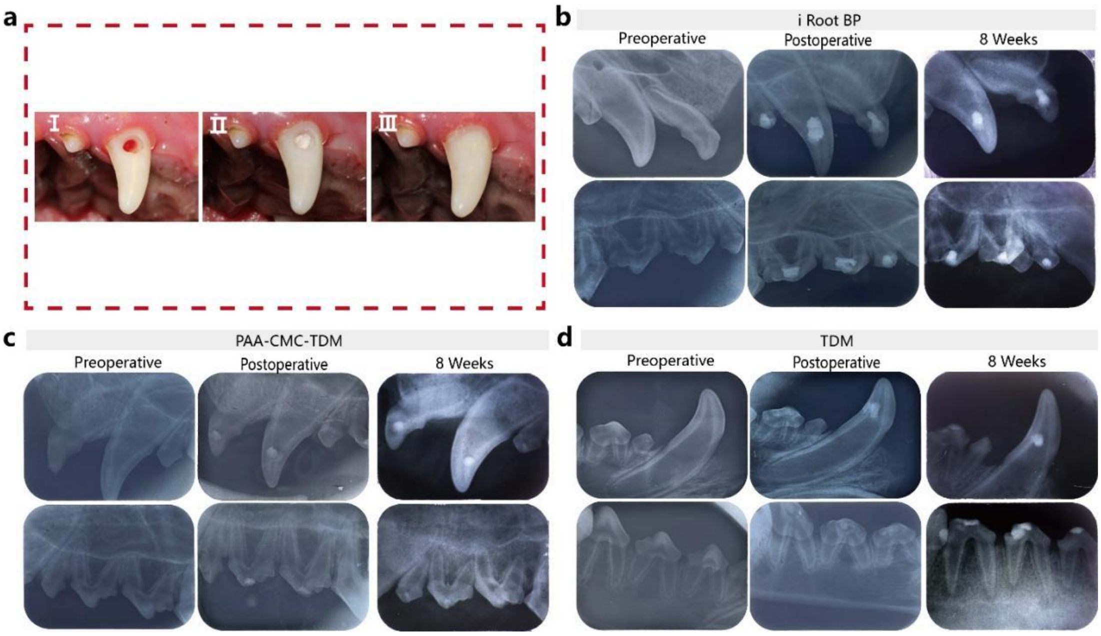

Supplementary Figure. 7 Construction of dental pulp defect model and X-ray images before, after and 8 weeks after direct pulp capping of Beagle dogs pulp repair model. **a** The preparation process of Beagle dogs dental pulp defect model, pulp cavity exposure (I), material filling (II), closure of the defect (III). **b** i Root BP plus, **c** PAA-CMC-TDM, **d** TDM. It can be found that the experimental tooth filling materials of each group were intact and not separated, and no obvious internal and external root absorption and other abnormalities were found.

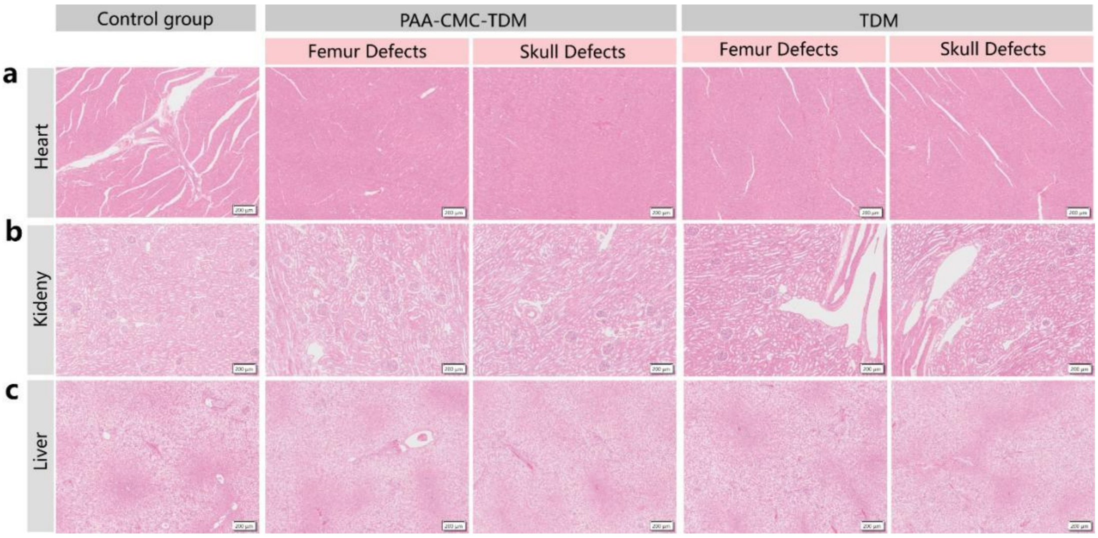

Supplementary Figure 8 HE staining of visceral tissues and organs about PAA-CMC-TDM implanted into the skull and femur defects of rats for 6 weeks. **a** HE staining of myocardial tissue. **b** HE staining of kidney tissue. **c** HE staining of liver tissue. Scale bar, (a, b, c): 200 μm.

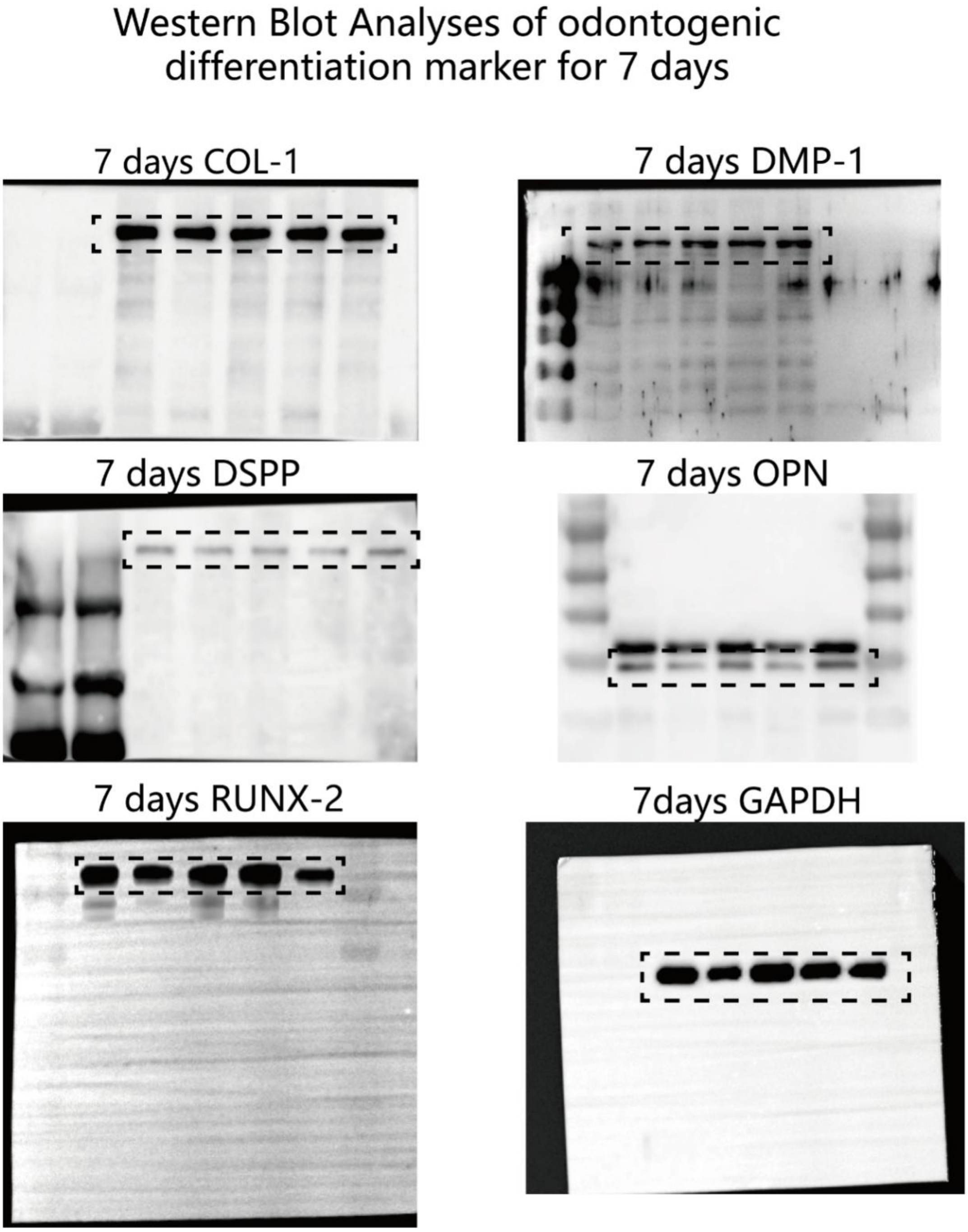

Supplementary Figure 9 The uncropped scans of the western blot Analyses of odontogenic differentiation marker blots for 7 days.

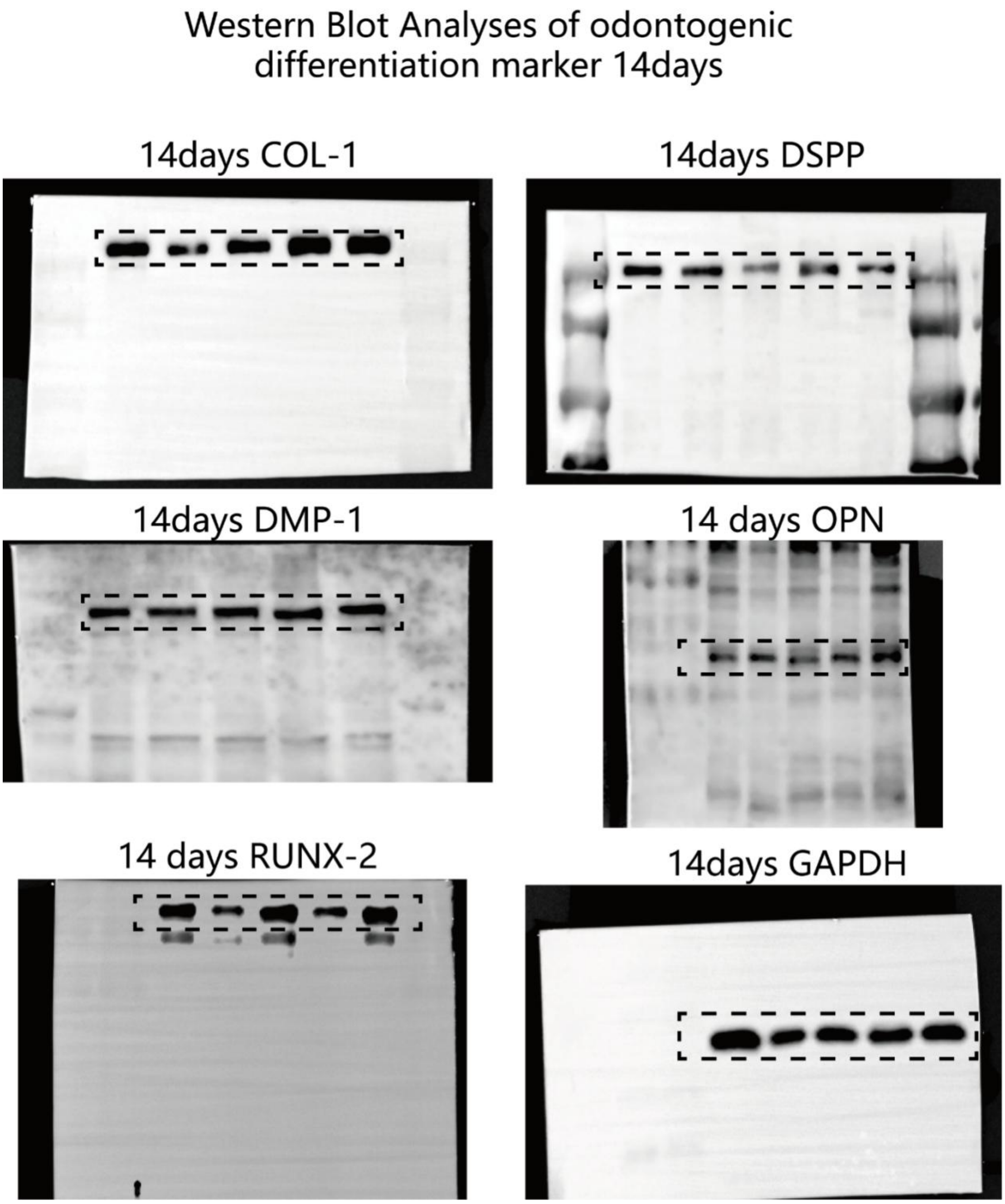

Supplementary Figure 10 The uncropped scans of the western blot Analyses of odontogenic differentiation marker blots for 14 days.

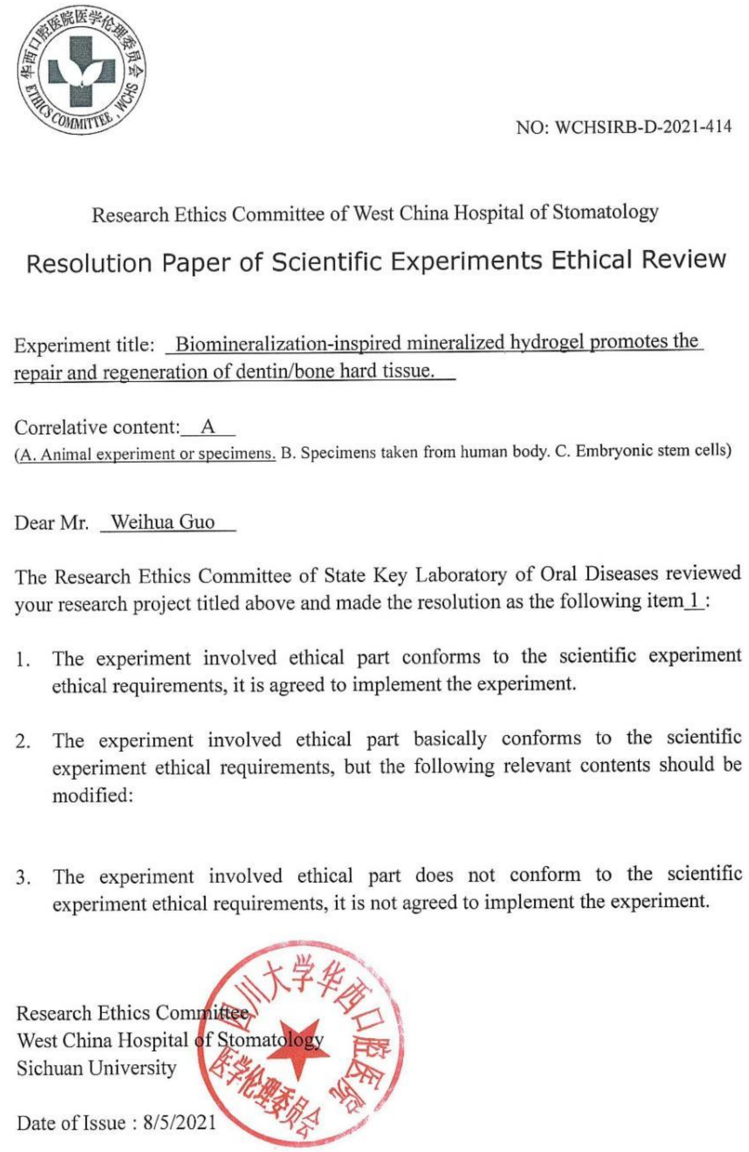

NO: WCHSIRB-D-2021-414

Research Ethics Committee of West China Hospital of Stomatology

Resolution Paper of Scientific Experiments Ethical Review

Experiment title: Biomineralization-inspired mineralized hydrogel promotes the repair and regeneration of dentin/bone hard tissue.

Correlative content: A

(A. Animal experiment or specimens. B. Specimens taken from human body. C. Embryonic stem cells)

Dear Mr. Weihua Guo

The Research Ethics Committee of State Key Laboratory of Oral Diseases reviewed your research project titled above and made the resolution as the following item 1 :

1. The experiment involved ethical part conforms to the scientific experiment ethical requirements, it is agreed to implement the experiment.
2. The experiment involved ethical part basically conforms to the scientific experiment ethical requirements, but the following relevant contents should be modified:
3. The experiment involved ethical part does not conform to the scientific experiment ethical requirements, it is not agreed to implement the experiment.

Research Ethics Committee
West China Hospital of Stomatology
Sichuan University

Date of Issue : 8/5/2021

Supplementary Figure 11 The West China Hospital of Stomatology Institutional Review Board, WCHSIRB-D-2021-414.

IRB WEST CHINA HOSPITAL of STOMATOLOGY Sichuan University

Institutional Review Board

West China Hospital of Stomatology
Sichuan University
No.14, 3rd Section, Renminnan Rd.
Chengdu, Sichuan 610041, China
Telephone: +86-28-85503684
Facsimile: +86-28-85503684
E-mail: hxkqlunli@163.com

To: **Weihua Guo**
West China Hospital of Stomatology, Sichuan University

Study Title:
Biomineralization-inspired mineralized hydrogel promotes the repair and regeneration of dentin/bone hard tissue

Approval Date: *8/5/2021* **Expiration Date of Approval:** *8/4/2022*

Submission type: Initial **Approval type:** Quick review

IRB Reference Number: WCHS-IRB-CT-2021-362 *(Please quote this ref # on all correspondence)*

Following files were reviewed:

Ethical Application Form	20210805
Protocol	20210805
Consent Form	20210805
Participant Information Sheet	20210805

The West China Hospital of Stomatology Institutional Review Board (WCSHIRB) has formally APPROVED this projects for the period indicated, in accordance with regulations or guidelines quoted below:

1. Quality Management Regulation for Good Clinical Practice By Food and Drug Administration, PRC 2003
2. Ethical Approval Review Methodology for Biomedical Research, PRC 2007
3. Management Regulation for Medical Technology Application By Ministry of Public Health, PRC 2009
4. The principle for ethical review of clinical trail. By Food and Drug Administration, PRC 2010
5. The Declaration of Helsinki(2008)

Investigators' Responsibilities:

If you are unable to complete the research activities before the expiration date, it is the Principal Investigator's responsibility to submit an application for extension and obtain approval before the expiration date. The written application for extension should be received by the WCSHIRB at least 30 days before the expiration date.

Any changes to any aspect of this study should be reported to IRB and obtain IRB approval before they can be implement. Any unanticipated problem, incompliance to protocol serious adverse event, should be reported to IRB.

四川大学华西口腔医院 医学伦理委员会

Yours sincerely

Chairmen of WCSHIRB

Wei Li

Supplementary Figure 12 The West China Hospital of Stomatology Institutional Review Board, WCHSIRB-CT-2021-362

Proof of copyright(

1. The copyright of this cover image belongs to Hangzhou Drawing Scientific Research Co., LTD and the author of the article(Bo Wen, Yuguo Dai, Xue Han, Fangjun Huo, Li Xie, Mei Yu, Yuru Wang, Ning An, Zhonghan Li, Weihua Guo) ownership.
2. Without the permission of the author, the content of this picture shall not be reproduced, otherwise it will be regarded as infringement.
3. Please indicate the source and original author when reprinting or quoting the content of this picture.
4 For those who do not abide by this statement or otherwise use the content of this picture illegally, the company and the author reserve the right to investigate according to law.

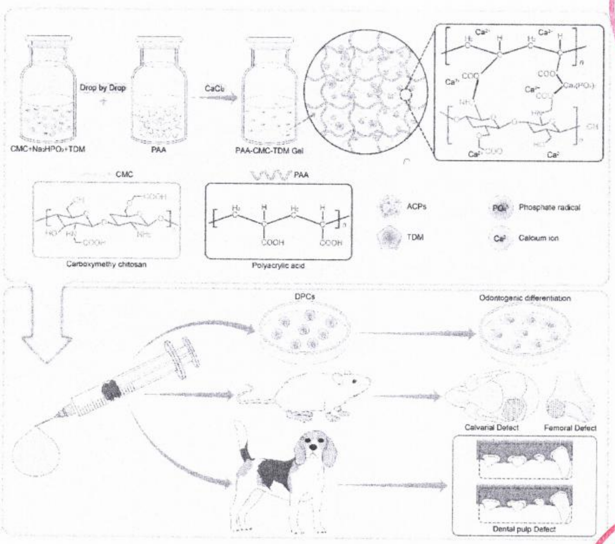

Supplementary Figure 13 The Proof of copyright for the Figue 1.
