# Supplementary material for: Biomineralization-inspired mineralized hydrogel promotes the repair and regeneration of dentin/bone hard tissue
Source: NPJ Regen Med. 2023 Feb 25;8:11. doi: 10.1038/s41536-023-00286-3 (PMC9968336; doi:10.1038/s41536-023-00286-3)
Supplement: Supplementary file 2 — Reporting Summary [file 41536_2023_286_MOESM2_ESM.pdf]

nature portfolio

Corresponding author(s): Weihua Guo

Last updated by author(s): Jan 28, 2023

Reporting Summary

Nature Portfolio wishes to improve the reproducibility of the work that we publish. This form provides structure for consistency and transparency in reporting. For further information on Nature Portfolio policies, see our Editorial Policies and the Editorial Policy Checklist.

Statistics

For all statistical analyses, confirm that the following items are present in the figure legend, table legend, main text, or Methods section.

n/a	Confirmed	
☐	☒	The exact sample size (*n*) for each experimental group/condition, given as a discrete number and unit of measurement
☐	☒	A statement on whether measurements were taken from distinct samples or whether the same sample was measured repeatedly
☐	☒	The statistical test(s) used AND whether they are one- or two-sided<br>*Only common tests should be described solely by name; describe more complex techniques in the Methods section.*
☐	☒	A description of all covariates tested
☐	☒	A description of any assumptions or corrections, such as tests of normality and adjustment for multiple comparisons
☐	☒	A full description of the statistical parameters including central tendency (e.g. means) or other basic estimates (e.g. regression coefficient) AND variation (e.g. standard deviation) or associated estimates of uncertainty (e.g. confidence intervals)
☒	☐	For null hypothesis testing, the test statistic (e.g. *F*, *t*, *r*) with confidence intervals, effect sizes, degrees of freedom and *P* value noted<br>*Give P values as exact values whenever suitable.*
☒	☐	For Bayesian analysis, information on the choice of priors and Markov chain Monte Carlo settings
☒	☐	For hierarchical and complex designs, identification of the appropriate level for tests and full reporting of outcomes
☒	☐	Estimates of effect sizes (e.g. Cohen's *d*, Pearson's *r*), indicating how they were calculated

Our web collection on statistics for biologists contains articles on many of the points above.

Software and code

Policy information about availability of computer code

Data collection	No software about commercial,open source and custom code was used to collect the date
Data analysis	Experimental data statistics in this study were performed using GraphPad Prism v8.0 and Origin 2019, and obtained and presented as mean ± standard deviation. The differences between two and multiple groups were compared by two-tailed student’s t-test and one-way analysis of variance. A value of p < 0.05 was considered statistically significant.

For manuscripts utilizing custom algorithms or software that are central to the research but not yet described in published literature, software must be made available to editors and reviewers. We strongly encourage code deposition in a community repository (e.g. GitHub). See the Nature Portfolio guidelines for submitting code & software for further information.

Data

Policy information about availability of data

All manuscripts must include a data availability statement. This statement should provide the following information, where applicable:

- Accession codes, unique identifiers, or web links for publicly available datasets
- A description of any restrictions on data availability
- For clinical datasets or third party data, please ensure that the statement adheres to our policy

All data, models, and code generated or used during the study are available from the corresponding author upon reasonable request.

Human research participants

Policy information about studies involving human research participants and Sex and Gender in Research.

Reporting on sex and gender

This information has not been collected. Only the third molars extracted for medical reasons were collected in this study, and gender was not reported to affect the results of this study or similar studies

Population characteristics

All the isolated teeth collected in this study were from healthy adults and had no underlying diseases.

Recruitment

The human dental pulp stem cells (hDPCs) and human-TDM material were derived from healthy third molars extracted for clinical reasons at West China Hospital of Stomatology under the guidelines of the West China Hospital of Stomatology Institutional Review Board after taking informed consent from all donators (WCHS-IRB-CT-2021-362).The collection plan of research samples can be designed according to patients' opinions and medical conditions.

Ethics oversight

the West China Hospital of Stomatology Institutional Review Board

Note that full information on the approval of the study protocol must also be provided in the manuscript.

Field-specific reporting

Please select the one below that is the best fit for your research. If you are not sure, read the appropriate sections before making your selection.

- [x] Life sciences
- [ ] Behavioural & social sciences
- [ ] Ecological, evolutionary & environmental sciences

For a reference copy of the document with all sections, see nature.com/documents/nr-reporting-summary-flat.pdf

Life sciences study design

All studies must disclose on these points even when the disclosure is negative.

Sample size

In the previous study, we determined the corresponding sample size based on the empirical method, which was confirmed by the Ethics Committee.
We performed the post-hoc effective size(w) calculation with SPSS software and power analysis using G-Power software.
In the femur defect model
For percent bone volume/tissue volume after 3 weeks of implantation of mineralized hydrogels, η2=0.815, α=0.05, power=28.86%
For percent bone volume/tissue volume after 6 weeks of implantation of mineralized hydrogels, η2=0.778, α=0.05, power=26.59%
for trabecular number after 3 weeks of implantation of mineralized hydrogels, η2=0.559, α=0.05, power=15.53%
for trabecular number after 6 weeks of implantation of mineralized hydrogels, η2=0.665, α=0.05, power=20.36%
In the cranial defect model
For percent bone volume/tissue volume after 3 weeks of implantation of mineralized hydrogels, η2=0.905, α=0.05, power=34.78%
For percent bone volume/tissue volume after 6 weeks of implantation of mineralized hydrogels, η2=0.794, α=0.05, power=27.56%
for trabecular number after 3 weeks of implantation of mineralized hydrogels, η2=0.731, α=0.05, power=23.87%
for trabecular number after 3 weeks of implantation of mineralized hydrogels, η2=0.930, α=0.05, power=36.52%
Taking one-way ANOVA P value, Tukey's multiple comparisons, and the post-hoc effective size(η2) into overall consideration, it represents differences among groups to some extent. In addition, other experimental results can be supportive of the bone repair outcome presented in this micro-CT analysis.
And for evaluating the ability of PAA-CMC-TDM to promote reparative dentin formation and explore its possibility for vital pulp preservation, we compared the hard tissue induction abilities of TDM, PAA-CMC-TDM and i Root BP plus using the Beagle dog dental pulp defect model. The i Root BP plus is a kind of mature commercial pulp capping material, so it serves as a positive control group.

Data exclusions

No data were excluded from the analyses.

Replication

We confirm that all attempts at replication were successful.

Randomization

This is not relevant to our study. All the experimental animals in this experiment are ordered from relevant animal companies with professional qualifications to ensure their controllable consistency. The feeding conditions and sampling time of animals are uniformly designed according to the requirements of the experiment, so as to avoid other interference factors as far as possible.

Blinding

During data collection and/or analysis, the researchers operate the relevant samples and analyze the data according to the unified standard, and the results obtained are objective and accurate. The result data in this study are objective statistics and pictures, in which the related differences can be observed and counted intuitively.

Reporting for specific materials, systems and methods

We require information from authors about some types of materials, experimental systems and methods used in many studies. Here, indicate whether each material, system or method listed is relevant to your study. If you are not sure if a list item applies to your research, read the appropriate section before selecting a response.

Materials & experimental systems

n/a	Involved in the study
☐	☒ Antibodies
☐	☒ Eukaryotic cell lines
☒	☐ Palaeontology and archaeology
☐	☒ Animals and other organisms
☒	☐ Clinical data
☒	☐ Dual use research of concern

Methods

n/a	Involved in the study
☒	☐ ChIP-seq
☒	☐ Flow cytometry
☒	☐ MRI-based neuroimaging

Antibodies

Antibodies used

Anti-Alkaline Phosphatase (ALP) antibody
HUABIO (China). ET1601-21 Anti-Alkaline Phosphatase Recombinant Rabbit Monoclonal Antibody [SA40-00]

anti-RUNX-2 antibody,
HUABIO (China). ET1612-47 Anti-RUNX2 Recombinant Rabbit Monoclonal Antibody [SD208-0]

Anti-osteopontin (OPN) antibody
HUABIO (China). 0806-6 Anti-Osteopontin Rabbit Polyclonal Antibody

Anti-type I collagen (COL-I) antibody
HUABIO (China). ET1609-68 Anti-COL1A1/Collagen I Recombinant Rabbit Monoclonal Antibody [ST58-04]

GAPDH
HUABIO (China). ET1601-4 Anti-GAPDH Recombinant Rabbit Monoclonal Antibody [SA30-01]

Anti-DMP-1
Novus Biologicals (USA). NBP1-45525 Synthetic peptide corresponding to residues surrounding amino acids 21 of rat DMP-1 (EDL99499).

Anti-CD68 antibody,
Abcam (UK). ab125212 Rabbit polyclonal to CD68

Vimentin antibody
Abcam (UK). ab92547 Rabbit monoclonal [EPR3776] to Vimentin - Cytoskeleton Marker

Anti-CD163 antibody
Abcam (UK). ab182422 Rabbit monoclonal [EPR19518] to CD163

Anti-DSPP rabbit pAb,
Zen Bioscience (Chengdu, China). 508413

Goat anti-rabbit IgG-HRP,
Zen Bioscience (Chengdu, China). 511203 Secondary Antibody

Goat anti-mouse IgG-HRP
Zen Bioscience (Chengdu, China). 511103 Secondary Antibody

Alexa FluoR 488 Goat anti-Mouse
Invitrogen (USA). A31627 Alexa Fluor™ 488 Goat Anti-Rabbit SFX Kit

Alexa FluoR 555 Goat anti-Rabbit
Invitrogen (USA). A32727 Goat anti-Mouse IgG (H+L) Highly Cross-Adsorbed Secondary Antibody, Alexa Fluor™ Plus 555

Validation

Anti-Alkaline Phosphatase (ALP) antibody
Predicted band size: 57 kDa Species: Human, Mouse, Ra Application: WB, IF-Cell, IHC-P, IP, FC

anti-RUNX-2 antibody,
Predicted band size: 57 kDaSpecies: Human, Mouse, Rat Application: IF-Cell, IF-Tissue, IHC-P, WB

Anti-osteopontin (OPN) antibody
Predicted band size: 35 kDa Species: Human, Mouse, Rat Application: WB

Anti-type I collagen (COL-I) antibody
Predicted band size: 139 kDa Species: Human, Cow Application: WB, IHC-P

GAPDH

Predicted band size: 36 kDa Species: Human, Mouse, Rat, Chicken, Zebrafish Application: WB, IF-Cell, IF-Tissue, IHC-P, FC, IP

Anti-DMP-1
Predicted band size: 130 kDa Species: Human, Mouse, Rat Application: ICC/IF, IHC, WB.

Anti-CD68 antibody,
Predicted band size: 75-110 kDa Species: Mouse, Rat Application: WB, IHC-P, IHC-Fr

Vimentin antibody
Predicted band size: 54 kDa Species: Mouse, Rat, Human, African green monkey Application: Flow Cyt (Intra), ICC/IF, WB, IHC-P

Anti-CD163 antibody
Predicted band size: 121 kDa Species: Mouse, Rat, Human Application: Flow Cyt, mIHC, IHC-P, WB, IHC-Fr
Anti-DSPP rabbit pAb,
Predicted band size: 130 kDa Species: Mouse, Rat, Human Application: WB

Goat anti-rabbit IgG-HRP,
Species: Rabbit Application: WB, IHC-P, ELISA

Goat anti-mouse IgG-HRP
Species: Mouse Application: WB, IHC-P, ELISA

Alexa FluoR 488 Goat anti-Mouse
Species: Rabbit Application: WB, ICC/IF

Alexa FluoR 555 Goat anti-Rabbit
Species: Mouse Application: WB, ICC/IF

Eukaryotic cell lines

Policy information about cell lines and Sex and Gender in Research

Cell line source(s)

The human dental pulp stem cells (hDPCs) were derived from healthy third molars extracted for clinical reasons at West China Hospital of Stomatology under the guidelines of the Ethical Review Committee after taking informed consent from all donators (Male/Female). The pulp tissue was minced with sterile scissors and subjected to enzymatic digestion with 1mg/mL type-Ⅰ collagenase for 30 min and tiled on the bottom of a culture bottle52. The DPCs were cultured in α-MEM supplemented with 10% FBS and 1% (v/v) penicillin/streptomycin solution at 37℃ in 5% CO2. The vimentin expression was analyzed by immunofluorescence staining following the manufacturer's protocol. Immunostaining was performed using primary antibodies against vimentin and the secondary antibody Alexa Fluor 488. After counterstaining the nuclei with the DAPI reagent, the cells were examined under a fluorescence microscope (Leica Optical, Germany). The hDPCs in passages 2–5 were used for the experiments.

Authentication

The vimentin expression of hDPCs was analyzed by immunofluorescence staining following the manufacturer's protocol. Immunostaining was performed using primary antibodies against vimentin and the secondary antibody Alexa Fluor 488. After counterstaining the nuclei with the DAPI reagent, the cells were examined under a fluorescence microscope (Leica Optical, Germany).
The cell lines used were not authenticated.We confirmed that the cells we obtained were derived from human dental pulp tissue, and had good value-added activity and multi-box differentiation ability

Mycoplasma contamination

We confirm that all cell lines tested negative for mycoplasma contamination

Commonly misidentified lines
(See ICLAC register)

The misidentified cell lines were not used in our study.

Animals and other research organisms

Policy information about studies involving animals; ARRIVE guidelines recommended for reporting animal research, and Sex and Gender in Research

Laboratory animals

The experimental protocol for the use of SD rats and Beagle dogs in this study was conducted in accordance with the approved guidelines set out by the Research Ethics Committee of West China Hospital of Stomatology (WCH-SIRB-D-2021-414). The female SD rats and female beagle dogs were obtained from Dashuo Experimental Animal Center (China). Animals were housed in ventilated cages on a 12:12-hour light/dark cycle with ad libitum access to food and water.

Wild animals

The study did not involve wild animals.

Reporting on sex

The sex of the animals selected in this study was unified as female in order to reduce the interference of other factors on the results. But in this study, gender is not an important grouping and experimental factor.

Field-collected samples	The study did not involve samples collected from the field.
Ethics oversight	Ethics approval and consent to participate<br>Research Ethics Committee of West China Hospital of Stomatology (No. WCHSIRB-D-2021-414， No.WCHSIRB-D-2021-362)

Note that full information on the approval of the study protocol must also be provided in the manuscript.
